# The Complete Genome Sequence of *Haloferax volcanii* DS2, a Model Archaeon

**DOI:** 10.1371/journal.pone.0009605

**Published:** 2010-03-19

**Authors:** Amber L. Hartman, Cédric Norais, Jonathan H. Badger, Stéphane Delmas, Sam Haldenby, Ramana Madupu, Jeffrey Robinson, Hoda Khouri, Qinghu Ren, Todd M. Lowe, Julie Maupin-Furlow, Mecky Pohlschroder, Charles Daniels, Friedhelm Pfeiffer, Thorsten Allers, Jonathan A. Eisen

**Affiliations:** 1 Department of Biology, Johns Hopkins University, Baltimore, Maryland, United States of America; 2 The Institute for Genomic Research (J. Craig Venter Institute), Rockville, Maryland, United States of America; 3 Institut de Génétique et Microbiologie, Université Paris-Sud, Paris, France; 4 Department of Biomolecular Engineering, University of California Santa Cruz, Santa Cruz, California, United States of America; 5 Department of Microbiology and Cell Science, University of Florida, Gainesville, Florida, United States of America; 6 Department of Biology, University of Pennsylvania, Philadelphia, Pennsylvania, United States of America; 7 Institute of Genetics, University of Nottingham, Nottingham, United Kingdom; 8 Department of Microbiology, Ohio State University, Columbus, Ohio, United States of America; 9 UC Davis Genome Center, University of California Davis, Davis, California, United States of America; 10 Department of Membrane Biochemistry, Max-Planck-Institute of Biochemistry, Martinsried, Germany; 11 Department of Biochemistry, University of Wisconsin-Madison, Madison, Wisconsin, United States of America; 12 Department of Medical Microbiology and Immunology, University of California Davis, Davis, California, United States of America; 13 Department of Evolution and Ecology, University of California Davis, Davis, California, United States of America; Miami University, United States of America

## Abstract

**Background:**

*Haloferax volcanii* is an easily culturable moderate halophile that grows on simple defined media, is readily transformable, and has a relatively stable genome. This, in combination with its biochemical and genetic tractability, has made *Hfx. volcanii* a key model organism, not only for the study of halophilicity, but also for archaeal biology in general.

**Methodology/Principal Findings:**

We report here the sequencing and analysis of the genome of *Hfx. volcanii* DS2, the type strain of this species. The genome contains a main 2.848 Mb chromosome, three smaller chromosomes pHV1, 3, 4 (85, 438, 636 kb, respectively) and the pHV2 plasmid (6.4 kb).

**Conclusions/Significance:**

The completed genome sequence, presented here, provides an invaluable tool for further *in vivo* and *in vitro* studies of *Hfx. volcanii*.

## Introduction

The moniker “model organism” is given to certain species if they have features that make them particularly useful for discovery of biological and biomedical principles. The features that make an organism particularly valuable as a model include ease of growth, availability of experimental tools (e.g., genetic manipulation), evolutionary relationship to other organisms of interest (e.g., human, crops, pathogens, etc.), and presence of interesting phenotypes.

One limitation in the use of model organisms is that they are very sparsely distributed across the tree of life. This is particularly true for the archaea [1]. Though this group represents one of the three main lines of descent in the tree of life, only a few species have been developed into what could be considered true model organisms. The limited number of model systems has presented a challenge for characterizing the biology of this key group of organisms. There are a number of reasons for this, not the least of which is the difficulty in growing many archaea.

However, there is one group of archaea for which many of the species are relatively easy to work with - the *Halobacteriaceae*. Though these species are obligate halophiles requiring high salt conditions to grow, because they are aerobic and mesophilic (they grow at moderate temperature), they can be grown in conditions much like those used for other model organisms such as the bacterium *Escherichia coli* and the model yeasts *Saccharomyces cerevisiae* and *Schizosaccharomyces pombe*. This ease of growth also makes *Halobacteriaceae* useful for introducing students to working with archaea since growing many other archaea requires expensive equipment or extensive training due to their thermophilic and/or anaerobic growth requirements.

The *Halobacteriaceae* form a monophyletic group within the phylum Euryarchaea of the domain Archaea. The *Halobacteriaceae* include 26 named genera (each with at least one cultured species - see http://www.the-icsp.org/taxa/halobacterlist.htm [2]: *Halobacterium* (abbreviated as *Hbt*.), *Haloadaptus, Halalkalicoccus, Haloarcula* (*Har*.), *Halobaculum, Halobiforma, Halococcus*, *Haloferax* (*Hfx*.), *Halogeometricum, Halomicrobium, Halopiger, Haloplanus, Haloquadratum* (*Hqr.*), *Halorhabdus, Halorubrum, Halosimplex, Halostagnicola, Haloterrigena, Halovivax, Natrialba, Natrinema, Natronobacterium*, *Natronococcus*, *Natronolimnobius*, *Natronomonas* (*Nmn*.), and *Natronorubrum*. As with most other groups of bacteria and archaea, many lineages are only known through rRNA-based studies of uncultured organisms (e.g., see [3]).

Of the haloarchaea, two very distinct species have been developed into models for experimental studies: the extreme halophile *Halobacterium* sp. NRC-1 (optimal NaCl 2.5–4 M) and the moderate halophile *Hfx. volcanii* (optimal NaCl 1.7–2.5 M). We note here that *Halobacterium* sp. NRC-1 and *Halobacterium salinarum* (*halobium*) refer to two very closely related isolates. Next to the different salt requirements, *Hbt.* sp. NRC-1 has some biological properties not found in *Hfx. volcanii* such as phototrophic growth employing rhodopsin-like proteins and the formation of gas vesicles. Conversely, *Hfx. volcanii* can degrade sugars such as glucose and synthesizes most of its amino acids. This not only allows for the study of archaeal carbohydrate utilization and amino acid biosynthesis, but also has proven to be highly useful for genetic selections etc. as *Hfx. volcanii* grows well on defined media. This is in contrast to *Hbt.* sp. NRC-1, which cannot degrade sugars and only synthesizes a minor subset of its amino acids leading, at least in part, to its rather poor growth on defined media. Hence both of these haloarchaea are highly valuable models to understand the diversity and ecology of high-salt environments but also to learn from their similarities and differences. Interestingly, both of these species of haloarchaea are highly polyploid [4].

While molecular biological and biochemical tools have been developed for both of these haloarchaea, the requirement for salinity close to saturation and the lack of a well-defined growth medium can interfere with *Hbt.* sp. NRC-1 *in vivo* assays. Moreover its highly mobile insertion elements cause frequent mutations [5], [6], [7]. In contrast, *Hfx. volcanii* grows on simple defined minimal media (either solid or liquid), accepts a wider range of relatively lower salt concentrations than most other extreme halophiles (including *Hbt.* sp. NRC-1) and its genome is significantly more stable [8].

Hence, over the past two decades this biochemically and genetically tractable moderate haloarchaeon has been invaluable in revealing insight into archaeal biology ranging from transcription to protein transport, modification and degradation. These studies have taken advantage of a diverse set of genetic, molecular and biochemical tools including among others, a simple knockout strategy [9], [10], [11], inducible promoters [12] and protein purification protocols, efficient, straight-forward transformation methods [13], [14], [15], shuttle vectors [16], [17], a diversity of selectable markers [1], beta-galactosidase [18] and short lived green fluorescent protein reporters [19], an ordered cosmid library [20] and genetic and physical maps [20], [21].

These tools have helped enable the use of *Hfx. volcanii* as a model for studies of various archaeal cellular processes such as protein transport [22], [23], [24], [25], protein glycosylation [26], lipid modification [27], tRNA processing [28], [29], gas vesicle formation [30], nucleotide synthesis [31], transcription [32], protein degradation [33], [34], [35], [36], DNA repair and recombination [37], [38], [39], [40], [41], [42] and DNA replication [43], [44].

Here we report on the sequencing of the genome of the type strain DS2. This strain was first described in 1975 [45] following its isolation from bottom sediment of the Dead Sea. It was initially known as *Halobacterium volcanii* (in reference to Benjamin Elazari Volcani who first demonstrated the existence of indigenous microbial communities in high salt environments [46]). We focused on the type strain to serve as a reference point for this species [47], and it is worth noting that strains WFD11 [48] and DS70 [49] are derived from DS2 and are widely used in the haloarchaeal community. This genome sequence, with proteome [50], [51], [52] and transcriptome [53] analyses in place, has been the missing piece in making this organism an outstanding model. Here we present analysis of this genome sequence in the context of previously obtained *in vivo* and *in vitro* work as well as the comparison of this sequence to four other haloarchaeal genomes.

We note that the genome sequence of this organism was made available a few years ago to the community in order to accelerate research and work on this organism. Using the genome data many new findings have been reported including but not limited to studies of the *agl* gene cluster [54], delineation of 3′ and 5′ UTRs [55], characterization of small RNAs [56], chromosomal replication [43], [57], RNA modification genes [58], and shotgun proteomics [51], [52]. While these studies have been enabled by the genome data, the lack of a publication describing the sequencing and analysis has been a hindrance. Thus in this paper we describe the sequencing and initial analyses of the genome.

## Results and Discussion

### I. General features of the genome and predicted genes

#### Genome features

The genome of *Hfx. volcanii* strain DS2 (Table 1) is composed of five circular genetic elements: a 2.848 Mb main chromosome, three smaller chromosomes (the 636 kb pHV4, the 438 kb pHV3 and the 85.1 kb pHV1) as well as 1 plasmid (the 6.35 kb pHV2). These results are generally consistent with the original genetic map of *Hfx. volcanii* [21]; the differences are discussed below. We use the term *chromosome* to describe the four largest elements because they all replicate using chromosome-like replication mechanisms [43] (see the following section) and we wish to preserve the previously published nomenclature—although we are aware that the terminology is under debate.

**Table 1:** General features of the *Hfx. volcanii* DS2 genome.

Feature	Main Chromosome	pHV4	pHV3	pHV1	pHV2	Total
Replicon size (bp)	2,847,757	635,786	437,906	85,092	6359	4,012,900
%GC content	66.6	61.7	65.5	55.5	56	65
rRNA operons	2	–	–	–	–	2
Number of tRNA	51	–	–	–	–	51
Number of CDSs	2949	638	381	89	6	4063
IS elements						
ISH51	16	22	1	6	–	45
IS4	8	15	1	6	–	30
Other	12	11	0	4	–	27
Total	36	48	2	16	–	102
Percent coding	86.6	82.9	85.5	80.8	79.5	–
Average pI of proteins	5.11	5.36	5.07	5.72	5.94	5.16

The average genomic GC content is 65%, with extensive variation found between and within replicons (see Table 1 and discussion below). The average coding density is 86%. The coding DNA of *Hfx. volcanii* is 65% GC, whereas non-coding DNA is 58% GC. GC bias is particularly extreme at the 3^rd^ codon position where it is 85% GC compared to 68% at the 1^st^ position and 44% at the 2^nd^ position.

#### Non-coding RNA genes

**Figure 1:**
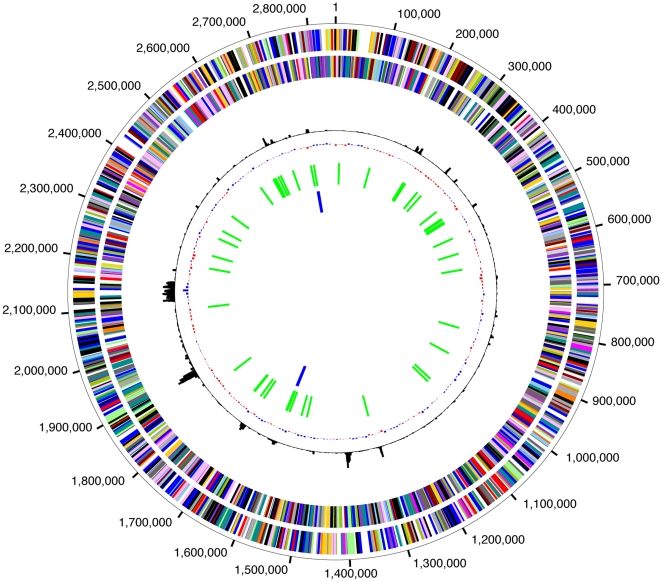
Circular graph of the chromosome of *Hfx. volcanii*. The circles display the following features, starting with the outermost circle: (1) forward strand CDSs; (2) reverse strand CDSs, (3) chi square deviation of local nucleotide composition from the genome average; (4) GC skew (G−C)/(G+C) (blue bars represent positive values, red bars represent negative values) (5) tRNAs (green lines); (6) rRNAs (blue lines). Gene color indicates the assigned role category. A gene can be included in the gene count for multiple role categories.

The genome encodes 6 rRNAs in two complete rRNA operons, two single-copy non-coding RNAs (RNase P and 7S RNA, which is the RNA component of the signal recognition particle [SRP], and 51 tRNAs that make up a complete functional complement (Figure 1). Notable among the tRNAs, there are three identical and adjacent pairs (Phe-GAA, Asp-GTC, Val-GAC) and three tRNAs that contain introns (Met-CAT, Gln-TTG, Trp-CCA). A single C/D box sRNA (which corresponds to the snoRNAs of eukaryotes) is present within the tRNA Trp intron and likely guides two positions of 2′-O-ribose methylation within the mature portion of tRNA Trp.

#### Protein-coding genes

In total, 4063 predicted proteins were identified in the genome. Of these, 226 (5.5%) did not have BLASTP matches to any protein entries in Genpept or to any proteins in any publicly available complete genome sequences (using an E-value cutoff of 0.01). Of the predicted coding segments (CDSs), 82.0% start with an ATG, 17.8% with a GTG, and 0.2% with a TTG.

#### Insertion sequences

Insertion sequences (IS) found include two major groups, both of which are members of the IS4 family: ISH51 [59] and a group of previously un-described IS4-type elements, which have been annotated as “IS4.” In addition, there are 27 IS elements of indeterminate classification. These elements are distributed unequally among the chromosomes. The main chromosome hosts 16 copies of ISH51 and 8 copies of the new IS4 element. pHV4, although only 22% of the length of the main chromosome, has more IS elements than any other replicon with 22 copies of ISH51, 15 copies of the new IS4 element, and 11 other IS-like elements. The small pHV1 also harbors a high number of IS elements for its size, with 6 copies each of ISH51 and the new IS4, plus 4 copies of unclassified IS elements. Chromosome pHV3 has few IS elements, and plasmid pHV2 has none.

#### Origins of replication

Recently, the origins of replication in *Hfx. volcanii* were experimentally characterized, and were analyzed in the context of genome data from this project [43]. We note those findings, which are of particular relevance to our genome sequence analysis here and discuss one key discrepancy between the genome analysis and experimental study.

The experimental work indicated that the main chromosome and the three smaller chromosomes (pHV4, pHV1, and pHV3) all contain functional autonomously replicating sequences (ARS). Unlike bacteria and most other archaea the experiments indicated that *Hfx. volcanii* has two replication origins on its main chromosome, *oriC*1 and *oriC2*. We note that the two rRNA operons (*rrnA* and *rrnB*) are quite close to these two origins on the main chromosome, with *rrnB* 6 kb from *oriC2* and *rrnA* 200 kb from *oriC1*. A similar pattern of rRNA-origin proximity has been noted in some bacterial genomes and is thought to allow some genes (*e.g.*, rDNA) to be transcribed more frequently during rapid growth [60]. It has also been hypothesized that the *rrn* operon closest to the origin (in this case, *rrnB*) is involved in translation of ribosomal proteins [60]. Both of these *rrn* operons are oriented away from the origins, presumably to avoid collisions between the machineries for replication and rRNA transcription.

There is one significant discrepancy between the genome analysis and the experimental results reported [43]. pHV1 and pHV4 were shown by Southern blot to contain a similar origin of replication sequence in *Hfx. volcanii* strains WFD11, DS70, and DS2. However, in the final genome assembly for DS2 reported here, no such sequence was found within the pHV4 contig (strain DS2) (despite rigorous sequence assembly evaluation [see methods]). One possible explanation for this discrepancy is that deletion of this element might have occurred in pHV4 when strain DS2 was grown to produce DNA for sequencing. Given that pHV4 harbors almost half of the insertion sequences found in *Hfx. volcanii*, it is possible that the genome rearrangement leading to the deletion of the replication origin from pHV4 was mediated by IS element transposition.

**Figure 2:**
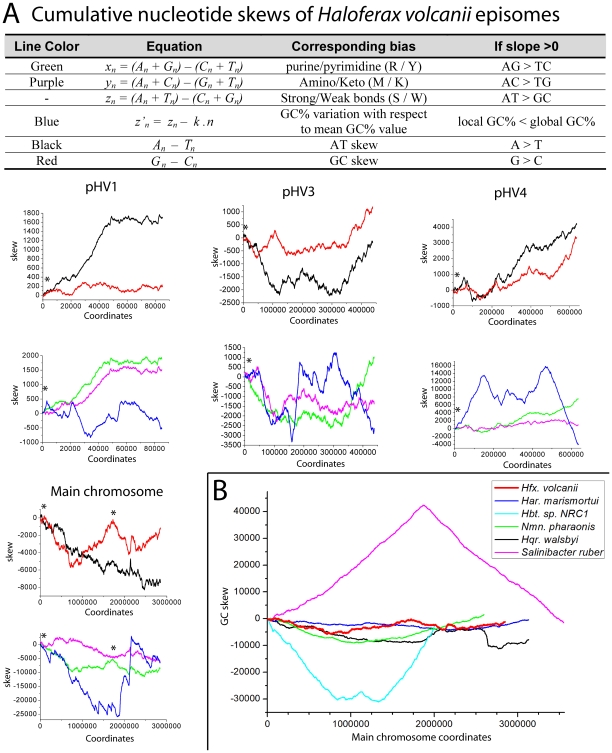
Nucleotide cumulative skews of *Hfx. volcanii* episomes and other halophilic bacteria and archaea. (A) Nucleotide skews obtained using the Zcurve method [61] for the four *Hfx. volcanii* chromosomes. The * indicates the positions of replication origins identified in [43]. (B) Comparison of the Zcurve GC skew from the main chromosome of *Hfx. volcanii* with those of other halophiles. The curves have been offset to start at respective replication origins and show the inverted GC skews of haloarchaea versus the halophilic bacterium that is characteristic of other bacteria and non-halophilic archaea as explained in [43].

Despite the apparent similarities in the current origins of replication between the main chromosome, pHV1, pHV3 and pHV4, our analysis suggests that there are both current and historical differences between these elements in either mechanisms of replication or location of origins. For example each presents different types of nucleotide composition skews (as detected with the Zcurve method [61]) (Figure 2A). These differences are interesting as skew is thought to be due to differential action of DNA polymerase for continuous (leading strand) and discontinuous (lagging strand) replication [62]. One possible cause of the different skew patterns would be use of different polymerase complexes for replicating the different genetic elements. This is in agreement with the identification of two different potential replicases, the DNA polymerases of the B and D families. DNA polymerases of the D-family are only found among euryarchaea. It has already been proposed that they could both participate in the DNA synthesis at the replication fork, the B-family polymerase for leading strand synthesis and the D-family for Okazaki fragments synthesis [63].

Comparison of the skew on the main chromosomes between species helps provide insight into the history of origins of replication in *Hfx. volcanii* (Figure 2B). One particular feature of note is that *Hfx. volcanii* has a relatively weak GC skew signal compared to other organisms and even other extreme halophiles. This pattern could be caused by the presence of two origins of replication on the chromosome, which might lead to conflicting skew pressures. Another (non mutually exclusive) hypothesis is that the origin(s) could have recently moved. Movement on the main chromosome origins could be catalyzed by genomic rearrangements by homologous recombination at the *rrn* operons, which is a common means of recombination in other species (*e.g.*, [64]). Consistent with this possibility, when comparing the genetic maps of *Hfx. volcanii* and the closely related *Hfx. mediterranei*, these *rrn* operons appear to be involved in a large inversion, one of the few genomic rearrangements seen between these species [8].

#### Comparison of the genome sequence with genetic maps

An initial genetic map of *Hfx. volcanii* was published in 1989 [21], followed by a more complete map in 1992 [65]. Many of the studies of *Hfx. volcanii* biology made during subsequent years made use of this map with its respective genetic markers and coordinates. The *Hfx. volcanii* genome sequence we present here is slightly smaller than originally estimated. The main chromosome, pHV4, and pHV3 sequences are 72, 54 and 4 kb smaller, respectively, than the values for the corresponding replicons from the genetic map. The two smallest genetic elements (pHV1 and pHV2) are in close agreement. Since we rigorously checked and re-evaluated the genome assembly (see Methods), we believe the discrepancies noted for the three largest replicons are most likely due to the errors inherent in estimating molecular weights for large DNA molecules by pulsed-field gel electrophoresis, the technique used to make the genetic map.

Two other small discrepancies between the genetic map and genome sequence were also identified. First, two tRNAs located on the map near ISH51 elements on pHV4 were not detected in the genome sequence. In the genome annotation presented here, all 51 tRNAs were located on the main chromosome. Second, the genetic map included a family of 43 ISH51 insertion elements distributed throughout the chromosomes and plasmid [59], [65], whereas the genome sequence identified three more, bringing the total to 46. The initial lower number is most likely due to the experimental limitations of the technique used, Southern blot hybridization.

#### Integrating the genetic map with the genome sequence

In order to relate the results of earlier, map-based studies of *Hfx. volcanii* to the genome data, we used the positions of the 24 genetic markers from the original genetic map paper (specifically we used table 3) [20] to superimpose the map onto the genome sequence annotation (see Text S1, Table S1). The genetic map was not detailed enough for larger genes and operons. Therefore, we did not include the markers for *RNase P*, *RNApol* subunits, and *trpA*, *D*, *E*, *F*, *G* because their inclusion would have diminished the ability to calculate an accurate conversion factor (see Text S1, Table S1). For the remaining 16 markers, the median distance was calculated from the marker's map position to the respective gene in the genome sequence. Since the resolution of the genetic map was only to the nearest 10 kb, the correspondence was resolved within 10 kb. Based on this approach, the coordinates in the annotated genome sequence are equivalent to 934.455 kb + x, where x is the position in the original 1989 genetic map.

One potential use for the integration of the genome and the genetic map of these species is in the re-interpretation of earlier studies that relied on the genetic map. We present one such analysis to reinterpret experimental studies by Ferrer *et al.* [66] and by Trieselmann and Charlebois [67] in Supplemental Information (see Text S1, Table S2 and S3).

### II: Comparison of genome structure and composition with other halophilic archaea

#### Genomic comparisons: Gene order vs. content differences

**Figure 3:**
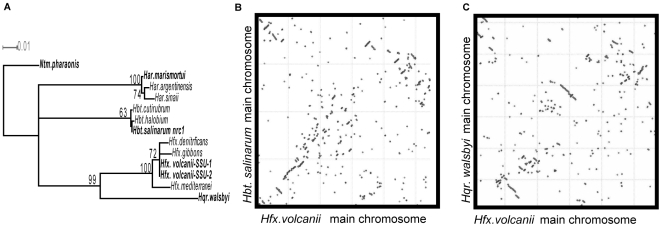
Phylogenomics of *Hfx. volcanii*. (A) 16S rRNA phylogeny of the haloarchaea that are closely related to *Hfx. volcanii* and that are discussed in the text. Organisms whose genomes are sequenced are in bold. (B and C) Genome alignments comparing the main chromosome of *Hfx. volcanii* with those of *Hbt.* sp. NRC-1 (B) and *Hqr. walsbyii* (C).

The current availability of five sequenced haloarchaeal genomes allows for robust, full genome comparisons. (Figure 3A). Comparison (as described in [68]) of the main *Hfx. volcanii* chromosome with those of the four other sequenced haloarchaea (*Hbt.* sp. NRC-1, *Har. marismortui*, *Nmn. pharaonis*, and *Hqr. walsbyi*) showed few long regions of alignment, although some shorter conserved regions were found between *Hfx. volcanii* and the two most closely related organisms, *Hbt.* sp. NRC-1 and *Hqr. walsbyii* (Figure 3B–C). Three-way comparisons showed that *Hfx. volcanii* shares 66% of its CDSs with *Hqr. walsbyi* and 63% with *Hbt.* sp. NRC-1 (Figure 3B).

#### GC content and amino-acid compositional biases

*Hfx. volcanii* has a highly biased nucleic acid and amino acid composition, phenomena that are common to most Halobacteriacea that have been sequenced. For example, GC content tends to be quite high (in *Hfx. volcanii* it is 65%). In addition, the average isoelectric point (pI) of the predicted proteome of *Hfx. volcanii* is 5.1, which is lower (more acidic) than the proteome of most organisms but is consistent with that observed in other haloarchaeal genomes. Multiple theories have been proposed to explain these biases, either separately, or jointly, as DNA and amino acid composition are interdependent.

One suggested explanation for the high GC content in halophilic archaea is that it is a result of selection to avoid thymidine dimers created by UV common to the solar environments where halophilic archaea are often found [69], [70]. However, this explanation is insufficient for three reasons: UV irradiation induces CC and CT dimers in addition to TT dimers [71], [72], many microbes exposed to high UV irradiation do not have high GC content [73] and some haloarchaea exposed to high solar irradiation have “normal” GC contents (*e.g.*, *Hqr. walsbyi* GC content is 48%) [74].

An alternative hypothesis proposed has been that the high GC content stabilizes nucleic acid base pairing in the face of high salt conditions [74]. Furthermore, these authors proposed that the lower GC content of *Hqr. walsbyi* is balanced by its requirement of high magnesium concentration for growth. However, the GC-salt stabilization theory appears insufficient for two reasons. First, Mojica *et al.* (1994) found that increasing the MgCl_2_ concentration from 1.5% to 30% led to a slight relaxation in *Hfx. volcanii* DNA topology, rather than an over-stabilization [75]. Second, if the hypothesis were true, the effect should be most pronounced in non-coding RNA (such as the structural RNA adaptations seen in thermophiles). However, the GC content of the rRNA of *Hqr. walsbyi* is higher than the average GC content of its genome and is closer to the GC content of the other haloarchaea.

**Figure 4:**
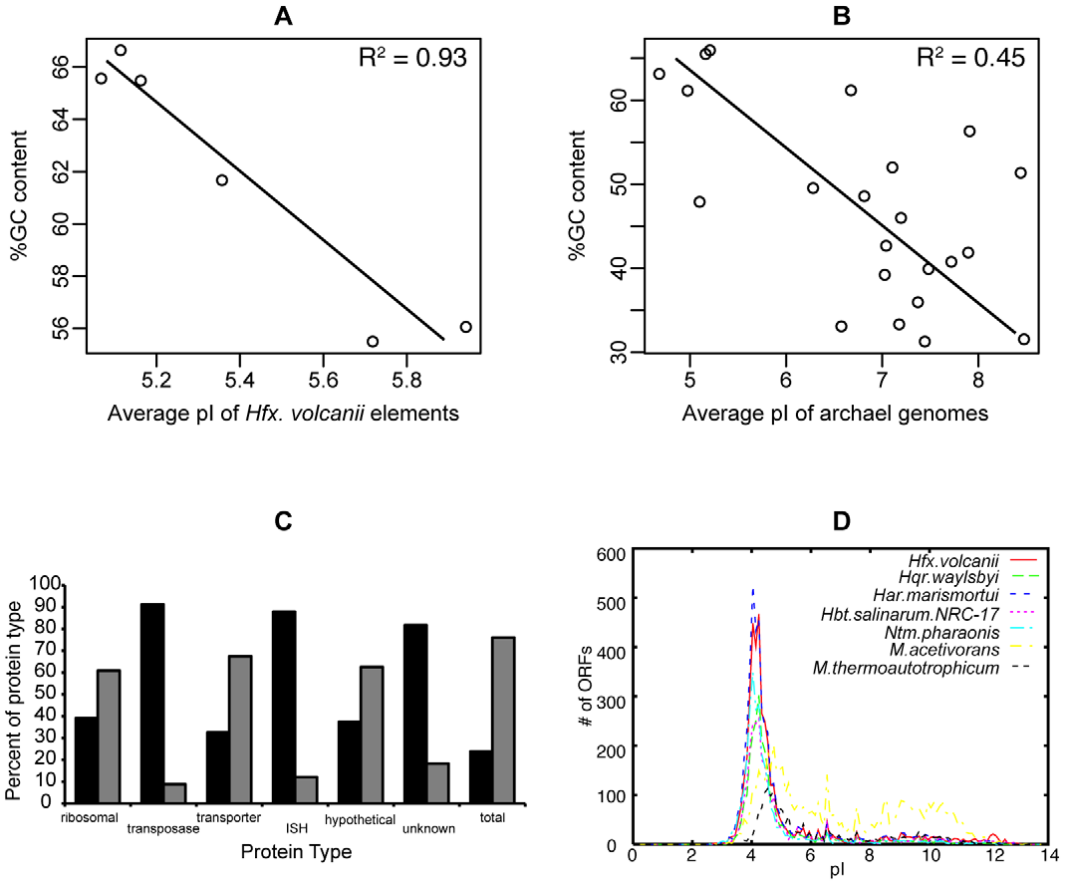
GC content patterns. Plot of the average %GC content of the genome vs. the average isoelectric point of the multiple chromosomes of *Hfx. volcanii* (A) and the predicted proteome for sequenced archaeal genomes (B). Groups of ORFs that show abnormal pI distribution (C). Distribution of pI of all proteins in haloarchaea compared to other archaea does not show a bimodal distribution (D).

A third possible explanation for the high GC content in haloarchaea is that it is an indirect result of selection for a highly acidic proteome. The opposite pattern, high AT content being correlated to a highly basic proteome, is well established in intracellular organisms (e.g., [76]). However, the indirect raising of the GC content by selection for acidic proteins seems unlikely in the haloarchaea as the codons used for acidic amino acids are not GC-rich. Furthermore, the only statistically significant pattern noted in the acidification of the proteome is an increase in use of aspartic acid and a decrease in lysine, neither of which contribute strongly to the GC content bias [77]. Other comparisons also suggest that there is no link between acidification of proteomes and increased GC content. For example, analyses focused on protein families known to have variable pIs only find weak correlation between pI and GC content across species [as in [78] and (data not shown)]. Although there is a strong relationship between pI and GC content when one compares the different genetic elements within *Hfx. volcanii* (Figure 4A), overall there appears to be no evidence of a causal connection between an acidic proteome and high GC content. Likewise, no significant correlation exists between genomic GC content and the average isoelectric point (pI) of the proteome for other archaeal species (Figure 4B).

**Figure 5:**
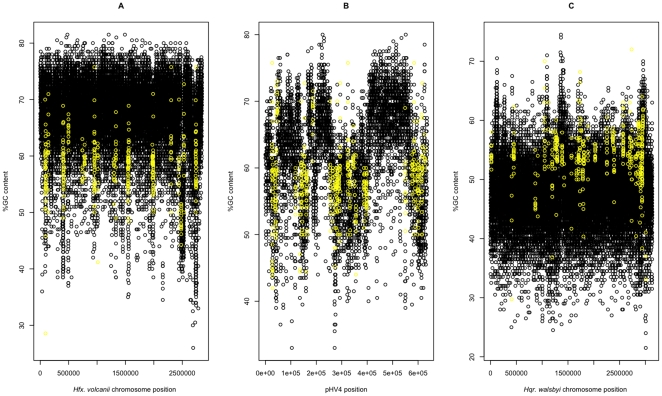
Haloarchaeal genomes are “evolving away” from IS elements in regard to GC content. Both the GC-rich *Hfx. volcanii* genome and the AT-rich *Hqr. walsbyi* genome are “evolving away” from their respective IS elements. Plot of GC content (black) overlaid with GC content of IS elements (yellow) for the *Hfx. volcanii* main chromosome (A), *Hfx. volcanii* pHV4 (B), and *Hqr. walsbyi* main chromosome (C).

Based on analysis of the *Hfx. volcanii* and other haloarchaeal genome sequences, we propose an alternative hypothesis that provides an explanation both for the high GC content observed in the haloarchaeal clade and the difference in GC content between non-coding (58% GC) and coding regions (65% GC) in *Hfx. volcanii*. Our hypothesis is that IS elements exert a selective pressure to avoid certain GC frequencies by having an insertion preference for a particular GC content. Furthermore we hypothesize that one can predict what the preference is from the GC content of the IS element itself. It is well known that transposable elements from across the tree of life including IS elements have preferences for regions of particular GC content. Thus, we propose that IS elements with an AT preference have driven the genomes and in particular the coding regions of haloarchaea to be of high GC content. Consistent with this hypothesis is the finding that the AT-rich ISH28 element preferentially transposes into GC-poor regions with little target sequence specificity in *Har. hispanica* [79]. In addition, analysis of the genome shows that AT-rich IS elements are “confined” to some degree to non-coding, AT-rich regions (Figure 5A–B), corroborating findings from genetic maps [65]. A similar finding was reported for *Hbt.* sp. NRC-1 [80].

This IS element avoidance hypothesis can explain patterns seen in the GC-rich genomes of *Hfx. volcanii*, *Har. marismortui*, *Hbt.* sp. NRC-1, and *Nmn. pharaonis*. It can also explain the situation in the GC-poor *Hqr. walsbyi* genome (48% GC) where the IS elements are higher GC content than the rest of the genome. The AT-rich *Hqr. walsbyi* genome, thus, can also be considered to be “running away from IS elements,” only it has run in the *opposite* direction, becoming more AT-rich than its IS elements (Figure 5C). Thus in our hypothesis, the key factor governing GC/AT composition is to avoid the composition that is ideal for IS activity. In fact, in order for *Hqr. walsbyi* to have become more AT-rich it presumably went through a period of “ideal composition” for IS insertion before it reached its current composition. Perhaps this period of “IS exposure” explains why there is less conservation of gene order between *Hqr. walsbyi* and *Hfx. volcanii* than between *Hfx. volcanii* and *Hbt.* sp. NRC-1 even though the former pair is more closely related.

#### Protein isoelectric point variation

As discussed above, GC content and pI of proteins appear to be somewhat if not completely independent in *Hfx. volcanii*. Nevertheless, there is evidence that there has been selective pressure on a highly acidic amino acid composition in haloarchaea and some halophilic bacteria (*e.g.*, *Salinibacter ruber* [81]). The majority of proteins in haloarchaea have an acidic exterior that increases the hydration shell of the folded protein and prevents “salting out”, thus enabling normal enzymatic function in a highly cationic environment. Based on analysis of the *Hfx. volcanii* genome a number of proteins were identified that were above average (more basic) in pI and, thus, may not have undergone this cellular adaptation. These included numerous transposases, proteins from IS elements, and proteins of unknown function (CDSs conserved in other species with currently uncharacterized function). In contrast, translation-associated proteins such as ribosomal proteins seemed to have become as acidic as possible (Figure 4C).

The proteome of many microbial genomes displays a bimodal distribution when plotted against pI [82]. Typically, cytosolic proteins are distributed around a peak at pI 6, whereas membrane-bound proteins cluster around pI 9. In *Hfx. volcanii* and the other haloarchaea, the bimodal pattern seems to have been outweighed by the strong selective pressure of the high salt environment. Here, the average pI for cytosolic proteins is 4.8, that for proteins containing a transmembrane domain (as determined by TMHMM prediction [83]) is slightly higher at 6.2. The whole proteome plot against pI shows only a single, strong peak around the proteome average of 5.1, demonstrating that the bimodal pattern is not universal (Figure 4D).

#### Codon usage variation

Synonymous codon usage (SCU) within a genome is usually homogenous, but local variation can result from selection, mutational bias, and DNA replication (*i.e.* nucleotide composition skew) [84], [85]. Lateral gene transfer (LGT) from other species can also introduce a local bias in the SCU, providing that the codon usage of the transferred genes has not yet adapted to that of the host [84], [86], [87], [88].

**Figure 6:**
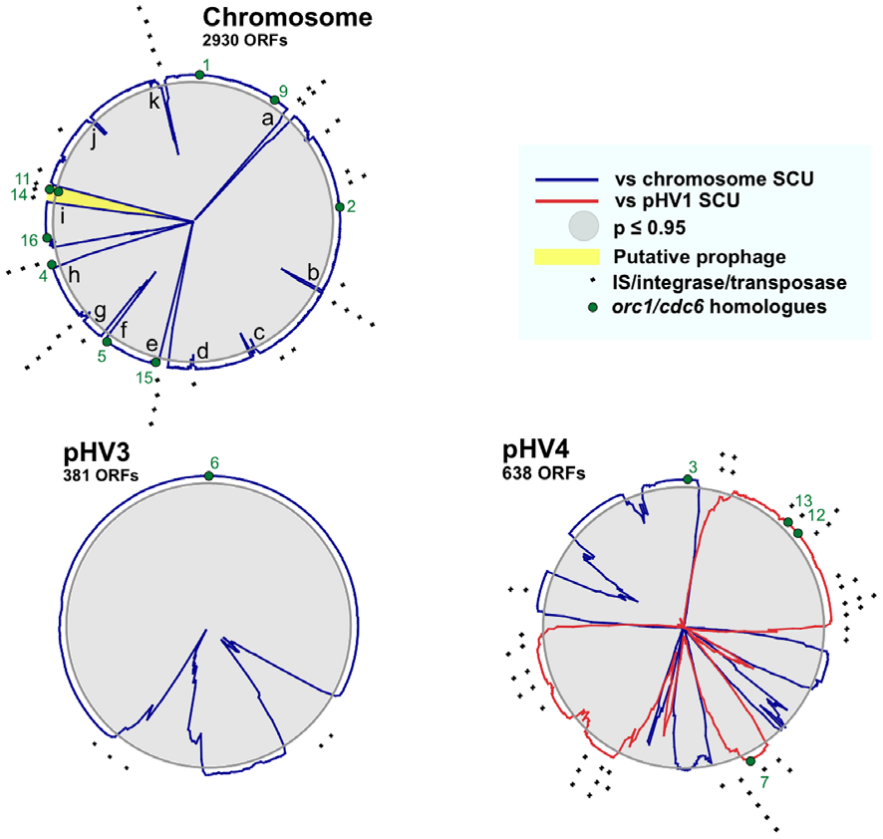
Codon usage in *Hfx. volcanii*. A synonymous codon usage (SCU) table was generated for each individual replicon. These were compared with SCU tables generated from a moving window of size of 30 protein coding sequences (CDS) along each replicon. Chi-square analysis (40 degrees of freedom, Stop, Met and Trp codons were omitted) was carried out to determine whether the SCU of each window of CDSs was statistically similar to each whole-replicon SCU. Comparisons were carried out in an automated manner using a C++ encoded program, SCUBA (Synonymous Codon Usage Bias Analysis).

On the main chromosome there are 11 genomic islands (GI) that display an unusual SCU, when compared to codon usage of the whole chromosome (Figure 6). These 11 GIs fall into two classes, depending on the presence of IS elements. Amongst the 4 GIs that do not contain IS elements (c, d, f, j), GI-f includes the second replication origin *oriC*2, the *orc5* initiator gene, and one of the two rRNA operons, *rrnB*. SCU bias in this region might result from proximity of a replication origin, or active transcription of *rrnB*. GI-j is also devoid of IS elements and contains 23 ORFs coding for ribosomal proteins, which are among the most highly expressed proteins in the cell. Thus, SCU bias might be due to selection of codon usage for optimal gene expression. The 7 remaining GIs are hotspots of IS elements and phage integrases, and might have been introduced by LGT and/or integrated plasmids. For example, genes encoding a restriction modification system are found in GI-i, which we suggest is a putative prophage (see section on DNA secretion system). Restriction modification systems are subject to frequent LGT and are often associated with mobility elements like phage [89].

pHV3 exhibits a similar SCU to the chromosome, apart from two GIs (Figure 6). All of the IS elements on pHV3 are found within or bordering these two GIs. By contrast, pHV1 has an SCU that is totally unlike the chromosome (data not shown). pHV1 contains many IS elements that are distributed evenly, suggesting that this entire replicon might have been acquired by LGT. pHV4 exhibits a chimeric SCU, with 6 GIs similar to the chromosome and 3 GIs with the same SCU as pHV1. The chimeric nature of pHV4 might be due to IS element-mediated DNA exchange between pHV4 and pHV1. Notably, most of the IS elements on pHV4 are located in or at the border of GIs with the same SCU as pHV1. Genomic rearrangement mediated by IS elements has been observed in *Hbt. salinarum* [6].

Frequent genetic exchange between pHV1 and pHV4 is underscored by the results of Norais *et al.* [43], which show that the DNA replication origin of pHV1 is also present on pHV4, while in the genome analysis presented here it is observed only on pHV1 (see section on origins of replication). Similarly, the region around the *orc3* gene, which was proposed by genome analysis to be the replication origin of pHV4, has been localized to the main chromosome [43].

It is noteworthy that 4 of the 9 *orc* genes on the main chromosome (*orc4*, *orc11*, *orc14*, and *orc15*) are found in GIs rich in IS elements. Furthermore, 3 of the 4 *orc* genes on pHV4 (*orc7*, *orc12* and *orc13*) are found in GIs with a SCU similar to pHV1. Interestingly, *orc13* is adjacent to a gene for the second B-family DNA polymerase (*polB2*), and together they are flanked by transposase genes. This supports the proposal made elsewhere that in archaeal species with multiple origins of replication, some of these origins and their associated replication proteins have been acquired by extrachromosomal element transfer [90], [91].

### III: Inferences about the biology of *Hfx. volcanii*

#### CRISPR elements

In 1995, Mojica *et al.* identified and characterized a 3 kb TREP (inverted Tandem REPeat) in *Hfx. volcanii* [92], now known as a CRISPR (Clustered Regularly Interspersed Short Palindromic Region) [93]. CRISPRs were initially proposed to be involved in chromosome partitioning and segregation. However, these regions in *Hfx. volcanii* and other microbial genomes are often associated with viral or transposable elements [94], and recent evidence suggests that CRISPR arrays may serve as an intracellular immune system, conferring resistance to phage [95].

The distribution of CRISPR regions varies within the haloarchaea, and likewise their number and location when present within a genome (Table 2). Notably, neither *Hqr. walsbyi* or *Hbt.* sp. NRC-1 contain CRISPRs or the CRISPR-associated genes (*cas1*-*cas6*) [96], both of which are encoded by the other sequenced haloarchaeal genomes. Since they have been found in most of the sequenced archaeal genomes, their absence from these two genomes is striking. These repeat elements have been implicated in an RNAi–like mechanism for silencing viral elements [94], [95], [97]. If CRISPR does serve this purpose, then the two genomes lacking these elements could be expected to be more prone to phage infections.

**Table 2:** Location of CRISPR regions in selected haloarchaeal genomes.

Genome	Copies	Location	Cas genes	Genome size (Mb)
*Hfx. volcanii*	3	2385045-2386594 (Main Chromosome)	Cas 1–6	4.01
“		204975-207584 (pHV4)		
“		217812-21856 (pHV4)		
*Har. marismortui*	5	1128141(Chromosome)	Cas 1–6	4.27
“		1032 (pNG400)		
“		35013 (pNG400)		
“		46654 (pNG400)		
“		20071 (pNG300)		
*Nmn. pharaonis*	4	165989 (Chromosome)	Cas 1–6	2.75
“		1578997 (Chromosome)		
“		97469 (PL131)		
“		27 (PL23)		
*Hqr. walsbyi*	none	none	no	3.18
*Hbt*. sp. NRC-1	none	none	no	2.57

#### Transport processes

Because the various haloarchaea have different requirements for salt, carbon, nitrogen, and metabolites, one might expect a corresponding variation in their transport capabilities. However, the only significant differences observed within the haloarchaea were the expansion of the ABC transporter family within *Hfx. volcanii* and the presence of the PTS in *Hfx. volcanii*, *Har. marismortui*, and *Hqr. walsbyi* (see below). There are no major expansions or contractions in the other 16 transporter gene families present in the haloarchaea (www.membranetransport.org). Overall, the archaea are rather homogenous in transport capacity, the exceptions being the ABC transporters which vary between 9 and 91 and the amino acid-Polyamine-Organocation (APC) family which is expanded in the Crenarchaea. Below we discuss three major categories of transport related genes and processes: the phosphotransferase system, sodium/proton antiporters and ABC transporters.

**Figure 7:**
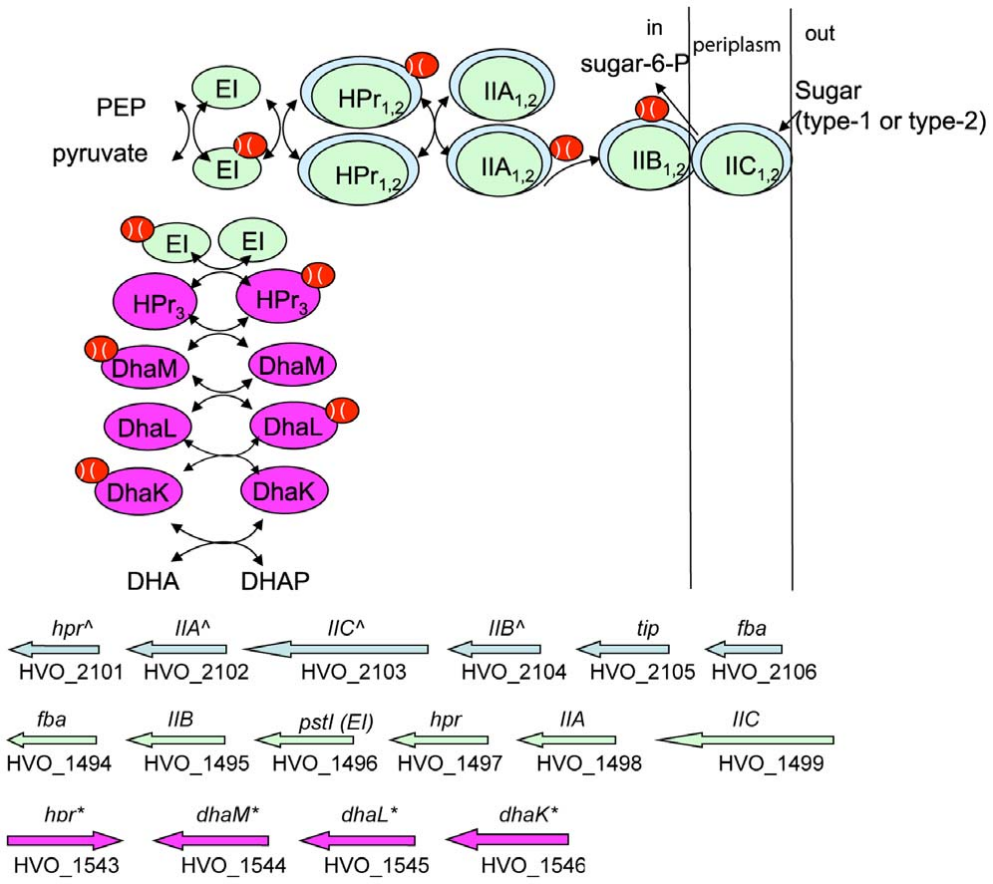
PTS system in *Hfx. volcanii*. (Top): a schematic diagram of the PTS system which is responsible for the concomitant transport and phosphorylation of sugar substrates and is highly regulatable (Bottom). The *Hfx. volcanii* genome contains a larger repertoire of the PTS system than previously reported in the archaea. * represents genes with homologs in the *Hqr. walsbyi genome*, *^∧^* represents genes with homologs in the *Har. marismortui* genome, the only other PTS genes described in archaea.

The phosphotransferase system (PTS) is an active sugar transport pathway found throughout the bacterial domain [98] but is only rarely found in archaea. The *Hfx. volcanii* genome encodes three predicted phosphotransferase systems (PTSs), each of which is encoded by a single operon (Figure 7). Two of these operons (HVO_2101-2106 and HVO_1494-1499) encode what are predicted to be complete membrane-bound enzyme II complexes consisting of IIA, IIB, IIC. The third operon (HVO_1543-1546) encodes a predicted cytosolic PTS complex, *i.e.*, the DhaK dihydroxyacetone kinase (DhaK/M/L) pathway. The genes in this latter operon are most similar to the PTS from bacteria and, thus, may represent a case of lateral gene transfer. To our knowledge, this is the first report of the complete Enzyme I/II/Hpr PTS in an archaeal genome. Previously, the non-membrane DHA DhaM/L/K-like PTS system was documented in the *Hqr. walsbyi* genome [74], but not the Enzyme I/II/Hpr PTS. Since PTSs are known to have numerous and diverse regulatory functions in bacteria [99], the presence of multiple PTSs in *Hfx. volcanii*—if functional—could allow for more complex regulated responses to a wide variety of environmental conditions. Along those lines, the third operon (HVO_2101-2106) is encoded within what appears to be a salt-sensitive transcribed region (see Text S1) and thus might have some role in osmoregulation, conceivably as a “complementation” for the lack of halorhodopsin in *Hfx. volcanii*, which is the otherwise conserved means of osmoregulation in all of the other haloarchaea to date.

Sodium/proton (Na^+^/H^+^) antiporters play a critical role in regulating both the Na^+^ level and pH in all domains of life, and are also thought to aid in adaptation to high salinity [100]. Some Na^+^/H^+^ antiporter genes found in the *Hfx. volcanii* genome are close relatives of other antiporters from *Euryarchaeota*. Interestingly, the Na^+^/H^+^ antiporter encoded by a seven gene operon (HVO_1063-1069) has no archaeal orthologs and is, instead, more closely related to the ubiquitous bacterial *Nha* Na^+^/H^+^ antiporter. This particular *Hfx. volcanii* antiporter is located adjacent to the *trk* potassium uptake operon and appears to be transcribed under high salt conditions (see Supplemental Text S1).

We found 69 predicted gene clusters for ABC transporters in the *Hfx. Volcanii* genome, which represents a broadly expanded repertoire in comparison to the first four other sequenced haloarchaea (Table 3) and the second highest number of ABC transporters found in any of the available sequenced archaeal genomes (see http://www.membranetransport.org). Part of this expansion can be attributed to the acquisition of three transporter systems (including both the binding protein and the permease domain) whose nearest orthologs are bacterial (HVO_A0297, 299-300, HVO_A0576-580, HVO_B0228-230). Furthermore, looking at the large ABC transporter superfamily, we found expansion of the ABC transporter families dedicated to sugar and peptide/oligopeptide/nickel transport. We hypothesize that this expansion is related to osmoregulation in *Hfx. volcanii* (see previous section). Although the importance of ABC oligopeptide and sugar transporters in adapting to salt stress has been documented in bacteria [101], their role has not yet been confirmed in the archaea.

**Table 3:** Homologs of ABC transporters, by COG category number, in selected haloarchaeal genomes.^1^

COG ID	Name	*H.v.*	*H.m.*	*H.s*	*N.p.*	*H.w.*
COG0395	ABC-type sugar transport system, permease component	10	7	1	0	1
COG0444	ABC-type dipeptide/oligopeptide/nickel transport system, ATPase component	12	2	2	1	3
COG0601	ABC-type dipeptide/oligopeptide/nickel transport systems, permease components	16	3	3	2	4
COG0614	ABC-type Fe3+-hydroxamate transport system, periplasmic component	13	9	5	2	3
COG0747	ABC-type dipeptide transport system, periplasmic component	14	2	3	3	3
COG3839	ABC-type sugar transport systems, ATPase components	12	8	1	0	1
COG3845	ABC-type uncharacterized transport systems, ATPase components	3	2	1	0	1
COG4603	ABC-type uncharacterized transport system, permease component	3	2	1	0	1
COG4608	ABC-type oligopeptide transport system, ATPase component	11	3	3	1	4

1 *H.v.*, *Haloferax volcanii*, *H.m.*, *Haloarcula marismortui*, *H.s.*, *Halobacterium* sp. NRC-1, *N.p.*, *Natronomonas pharaonis*, *H.w.*, *Haloquadratum walsbyi*.

#### Rnase R

*Hfx. volcanii* has the distinction of being the first—and so far the only—organism shown to not carry out RNA polyadenylation or exosome-like RNA degradation [102], [103]. Consistent with this, homologs of various yeast exosomal subunits (Rrp4 [COG1097], Rrp41 [COG0689], Rrp42 [COG2123], and Csl4 [COG1096]) as well as homologs of known poly(A) polymerases [104], [105] were not detected in the *Hfx. volcanii* genome sequence. These exosomal proteins are also absent from the other haloarchaea, suggesting that the entire clade lacks these capabilities.

As observed by Portnoy *et al.* (2006), RNase R is the “obvious candidate” for executing mRNA degradation in haloarachaea, and it has been shown to be required for viability and degradation of structured RNAs [103]. A phylogeny of the RNase R homologs in the haloarchaea shows that these genes are more closely related to bacterial RNase R (not shown) and, in fact, appear to be a sub-group within the bacterial group. This phylogenetic relationship is of particular interest because *in vitro* work suggests that this haloarchaeal enzyme is adapted to a mesophilic environment [103].

#### DNA repair and recombination

In this section we discuss the aspects of DNA repair and recombination genes and processes in *Hfx. volcanii*: DNA mismatch repair, homologous recombination and nucleotide excision repair.

DNA mismatch repair (MMR) has been extensively characterized in diverse bacteria and eukaryotes and has been shown to play several critical roles in safeguarding genomic information including correcting replication errors and regulating recombination rates and patterns [106], [107]. The general schema for MMR and the core proteins involved are conserved between bacteria and eukaryotes; all species studied that have MMR have been found to use homologs of the MutS and MutL proteins of *E. coli*. Surprisingly, when the first archaeal genomes were sequenced they were found to either not encode homologs of these proteins or to only encode highly divergent MutS-like proteins that apparently are not involved in MMR [108]. However, PCR experiments [40] and subsequent genome sequencing projects (e.g., [109]) revealed that species in the *Methanosarcinales* and *Halobacteriales* do encode homologs of MutS and MutL. Analysis of the genome reveals that *Hfx. volcanii* encodes four MutS and two MutL homologs. This pattern (multiple MutS- and MutL-like proteins) is common in eukaryotes where the different proteins have been found to have distinct roles, with some of the MutS homologs not being involved in MMR. Analysis of the predicted proteins in *Hfx. volcanii* supports the possibility of diverse functions. Two of the MutS homolog encoding genes are located in operons with a *mutL* partner, and their products belong to the MutS1 family that is involved in MMR in other species [108], [110]. Given that *Hfx. volcanii* does not have an elevated spontaneous mutation rate [111], we propose that it is likely to have MMR. The other two MutS homologs belong to the poorly-characterized MutS2 supergroup of which most of the characterized members do not function in DNA repair [108], [110], [112].

*Hfx. volcanii* encodes a RecA-family recombinase RadA [37], [113] which has previously been shown to be essential for homologous recombination and important for cell viability and repair of DNA damage [39]. Like other euryarchaea, *Hfx. volcanii* also encodes a RadA paralog known as RadB [114] RadB has been shown to act in DNA repair [41] and interacts with both RadA and the Holliday junction resolvase Hjc [115]. A gene for Hjc is found in *Hfx. volcanii*. Homologs of the DNA double-strand break repair proteins Mre11 and Rad50 are present. Interestingly deletion of these genes results in increased resistance to DNA damage [42].

Like most archaea, genes encoding a subset of the eukaryotic system for nucleotide excision repair (NER) are found in *Hfx. volcanii*. For example, a homolog of the eukaryotic Xpf is present, the euryarchaeal protein is termed Hef [116]. A homolog of eukaryotic Xpg is also found, however the archaeal protein is annotated as Fen1 and might act instead in DNA replication. In addition to these “eukaryotic” NER genes, homologs of the bacterial UvrABCD system for NER are found in *Hfx. volcanii*, as in related haloarchaea. Work in the related species *Halobacterium* sp. NRC-1 has shown that these proteins act in NER [117] and *Hfx. volcanii* does have an NER like process [40].

#### Restriction systems

Analysis of the genome provides some insight into the known DNA restriction properties of *Hfx. volcanii*. For example, it was noted as early as 1987 that DNA extracted from *Hfx. volcanii* is recalcitrant to cleavage by *Xba*I, *Spe*I, *Nhe*I, and other restriction enzymes that use a central CTAG tetranucleotide recognition site [48]. This protection may be provided by HVO_0794, which encodes a Zim CTAG modification methylase. It has also been noted that transformation of *Hfx. volcanii* is more efficient with DNA from *E. coli* strains with *dam* ^−^ mutations (which make the cell unable to methylate at GATC sites) than from *dam^+^* strains. This and other evidence suggests that *Hfx. volcanii* possesses a methylation sensitive GATC targeting restriction enzyme [16] which might be encoded by the putative Mrr-family endonuclease HVO_0682.

#### DNA replication

The core genes involved in DNA replication have been annotated and examined (Table 4), including a complete set of the genes considered to be required. Below we discuss five major categories of DNA replication related genes and processes: DNA polymerases, primases, RNAseH, DNA ligases, and DNA replication initiators.

**Table 4:** DNA replication associated genes in the *Hfx. volcanii* DS2 genome.

Function	Gene	Locus tag	Episome	Coordinates	COG	Specificity
Replication initiators (Cdc6/Orc1)	*orc1*	HVO_0001	Chromosome	258–1952	1474	Associated to *oriC1*
*“*	*orc2*	HVO_0634	Chromosome	569203–570429	1474	
*“*	*orc3*	HVO_A0001	pHV4	152–1402	1474	Associated to *oripHV4-2*
*“*	*orc4*	HVO_2042	chromosome	1888354–1889583	1474	
*“*	*orc5*	HVO_1725	Chromosome	1594707–1595912	1474	Associated to *oriC2*
*“*	*orc6*	HVO_B0001	pHV3	201–1433	1474	Associated to *oripHV3*
*“*	*orc7*	HVO_A0257	pHV4	258668–257391	1474	
*“*	*orc8*	HVO_C0057	pHV1	56369–57616	1474	
*“*	*orc9*	HVO_0194	Chromosome	174892–176016	1474	
*“*	*orc10*	HVO_C0001	pHV1	101–1327	1474	Associated to *oripHV1*/*4*
*“*	*orc11*	HVO_2293	Chromosome	2162076–2162873	1474	
*“*	*orc12*	HVO_A0072	pHV4	66289–65261	1474	
*“*	*orc13*	HVO_A0064	pHV4	56897–55455	1474	
*“*	*orc14*	HVO_2292	Chromosome	2161873–2160851	1474	
*“*	*orc15*	HVO_1537	Chromosome	1403626–1404828	1474	
*“*	*orc16*	HVO_2133	Chromosome	1998477–1999643	1474	
Helicase	*mcm*	HVO_0220	Chromosome	199368–201476	1241	
Single-strand DNA binding proteins	*rpa1*	HVO_1338	Chromosome	1218023–1219306	1599	
*“*	*rpa2*	HVO_0519	Chromosome	453269–454720	1599	
*“*	*rpa3*	HVO_0292	Chromosome	262126–261191	1599	
Primases	*priS*	HVO_2697	Chromosome	2544015–2545172	1467	Eukaryotic-type small catalytic subunit
*“*	*priL*	HVO_0173	Chromosome	155952–157163	2219	Eukaryotic-type large regulatory subunit
*“*	*dnaG*	HVO_2321	Chromosome	2187520–2188938	0358	Bacterial-like
Polymerases	*polD1*	HVO_0003	Chromosome	2942–4540	1311	D-family, DP1 subunit 3′-exonuclease
*“*	*polD2*	HVO_0065	Chromosome	64044–67649	1933	D-family, DP2 subunit
*“*	*polB*	HVO_0858	Chromosome	770335–774429	0417	B-family
*“*	*polB2*	HVO_A0065	pHV4	59942–57789	0417	B-family
*“*	*polX*	HVO_0741	Chromosome	660854–662599	1387	X-family
*“*	*polY*	HVO_1302	Chromosome	1187785–1186496	0389	Y-family
PCNA	*pcnA*	HVO_0175	Chromosome	157928–158671	0592	
PCNA loader	*rfcA*	HVO_0203	Chromosome	181286–182296	0470	RF-C small subunit
*“*	*rfcB*	HVO_2427	Chromosome	2295031–2296497	0470	RF-C large subunit
*“*	*rfcC*	HVO_0145	Chromosome	135168–136193	0470	RF-C small subunit
FLAP endonuclease	*fen1*	HVO_2873	Chromosome	2710411–2711391	0258	
Ligases	*ligA*	HVO_1565	Chromosome	1433356–1435113	1793	ATP-dependent
*“*	*ligN*	HVO_3000	Chromosome	2831155–2833254	0272	NAD-dependent
Topoisomerases	*topA*	HVO_0681	Chromosome	609107–611629	0550	type I
*“*	*top6A*	HVO_1570	Chromosome	1437643–1438743	1697	type VI, A subunit
*“*	*top6B*	HVO_1571	Chromosome	1438743–1441139	1389	type VI, B subunit
*“*	*gyrA*	HVO_1573	Chromosome	1443314–1445899	0188	type II, A subunit (gyrase)
*“*	*gyrB*	HVO_1572	Chromosome	1441393–1443312	0187	type II, B subunit (gyrase)
*“*	*rnhA*	HVO_2438	Chromosome	654302–654895	0328	type I
*“*	*rnhB*	HVO_1978	Chromosome	1824240–1824887	0164	type II
*“*	*rnhC*	HVO_A0463	pHV4	469311–469961	0328	type I
*“*	*rnhD*	HVO_A0277	pHV4	284563–285140	0328	type I - pseudogene
*“*	*ginS*	HVO_2698	Chromosome	2545169–2546164	1711	
*“*	*dna2*	HVO_2767	Chromosome	2607274–2609928	1468	

Note some of these identifications are based on functional studies, some are predictions based on analysis of the genome sequence.

 Five different DNA polymerases have been identified in the *Hfx. volcanii* genome. Two B-family polymerases (PolB) that are found in all archaea, one on the chromosome (HVO_0858) and one on pHV4 (HVO_A0065). Owing to the fact that the three eukaryotic replicases (Polα, Polδ and Polε) also belong to the B-family, this PolB is most probably implicated in genomic duplication in *Hfx. volcanii*. A D-family polymerase (PolD1 and PolD2) was also identified. This heterodimeric DNA polymerase is specifically found in Euryarchaea. The PolD1 subunit presents a 3′ to 5′ proofreading exonuclease activity [118]. The PolD2 subunit alone presents only a weak catalytic activity that is highly enhanced by the addition of the PolD1 subunit. The resulting holoenzyme possesses a strong polymerase/proofreading exonuclease activity indicating a possible role in genomic duplication conjointly with PolB [63]. Finally, *Hfx. volcanii* encodes a DNA polymerase of the X-family and the Y-family. Family-X enzymes are usually small DNA polymerases whose primary role is to fill gaps of one to a few nucleotides during DNA repair processes such as base-excision repair or double strand break repair [119]. Family-Y enzymes are error-prone (lesion bypass) polymerases and are homologous to the bacterial enzyme DinB.

The *Hfx. volcanii* genome encodes both the classical “eukaryotic-like” primase PriS/L and a distant homolog of the bacterial primase DnaG, the latter being nonessential [120]. The genome also encodes two types of RnaseH: the archaeal type II protein (*rnhB*) and a type I homolog probably of bacterial origin (*rnhA*). These proteins are thought to function in conjunction with the flap structure-specific endonuclease 1 (Fen1) in processing Okazaki fragments. The triple mutant, Δ*rnhA* Δ*rnhB* Δ*fen1*, was viable, indicating that their function in DNA replication is not essential for cell growth [121]. *Hfx. volcanii* encodes two DNA ligases: a NAD^+^-dependent protein (*ligN*, probably of bacterial origin) and the archaeal ATP-dependent enzyme (*ligA*). Cells with either gene inactivated are viable, but the double deletion is lethal [44].

Most archaea encode only one to three Cdc6/Orc1 initiator proteins that bind to the ORB sequences at *oriC*s, but the haloarchaea usually contain a large *cdc6/orc1* gene family with typically more than ten homologs. In *Hfx. volcanii*, 16 genes encoding Cdc6/Orc1 initiators were identified and at least one homolog was present on every chromosome. The phylogeny of the *cdc6/orc1* in the haloarchaea indicates that this gene family has undergone several relatively recent gene duplication events. With this many homologs present, it is likely that there is some amount of redundancy and/or divergence of function. Consistent with both of these possibilities, experimental deletion of three homologs (*orc1*, *orc5* and *orc10*) demonstrated that they are dispensable for cell growth under normal laboratory conditions, indicative of either redundancy of function within this gene family [43]. For example some might act as activators and others as repressors of replication initiation, as has been observed in *Sulfolobus* sp. [122]. The possibility of divergence of function is suggested by the large variation in pIs among the proteins (3.98 to 5.59). This could indicate that different homologs are individually adapted to cope with the varying intracellular KCl concentrations resulting from the cellular response to variable environmental salt concentrations.

#### Possible DNA secretion system

A 53-kb region in the *Hfx. volcanii* main chromosome between coordinates 2110297 and 2163576 presents several unique features such as a lower GC content (50.1% versus 66.7% for the rest of that chromosome) and a very high concentration of stop codons in the non-coding phases. This region encodes homologs of XerC/D recombinases as well as of VirB4/D4 (proteins implicated in type IV DNA secretion in bacteria. This region might encode a DNA secretion system that *Hfx. volcanii* could have adapted from a defective former prophage. Such systems have been reported for other archaea and bacteria, including *Bacillus subtilis* [123], *Neisseria gonnorrhoeae* [124], and *Methanococcus voltae* [125]. If present, this could explain the high transformation efficiency, natural competence, and mating ability of *Hfx. volcanii* [126] [127]. It can also provide a possible mechanism for the hypothesis that there has been significant amounts of lateral gene transfer between halophilic archaea and halophilic bacteria [81].

#### The secretome of *Hfx. volcanii*

The secretome of *Hfx. volcanii* includes a large number of proteins predicted to be secreted in a folded conformation via the twin-arginine translocation (Tat) pathway. This is consistent with previous findings that the haloarchaea, distinct from all other archaea and most bacteria, use this pathway extensively, possibly reflecting an adaptation to their high salt environments [22]. Many of the Tat substrates also have a lipobox motif and thus are likely to be lipid-anchored in the membrane as has been reported for *Nmn. pharaonis* [128], [129]. Nearly all of the ABC transporters substrate-binding proteins exemplify such a Tat-lipobox motif combination. In addition, we also identified a significant number of putative Sec substrates using SignalP [130], thus suggesting that some haloarchaeal proteins are secreted in an unfolded conformation.

Interestingly, use of FlaFind, a recently developed program capable of predicting Sec substrates with class III signal peptides, not only identified additional putative Sec substrates, but also revealed that many of the SignalP-positive substrates are in fact predicted to contain a class III Sec signal peptide [131]. Class III Sec signals are associated with bacterial and archaeal cell-surface structures, including bacterial and archaeal type IV pili as well as archaeal flagella [132]. Since folding of the subunit and its assembly into the cell-surface structures are likely co-dependent processes, translocation of the unfolded proteins via the Sec secretion system would allow assembly in the external environment. Consistent with this supposition, the genes encoding a significant number of these substrates are co-transcribed with homologs of pili/flagella biosynthesis genes. Three of these operons are conserved among several haloarchaea.

#### Bacterial-like cold shock proteins

The major cold-shock protein family CspA (and its homologs) (COG1278) is widespread in bacteria and eukaryotes, with multiple paralogs present in many organisms. Family members have RNA chaperone and transcription anti-termination activities, which enhance survival during cold-shock acclimation [133], [134]. Initial studies failed to identify members of this family in archaea [135]; however, genome sequencing and analysis revealed members of this family in halophilic archaea. In fact, each haloarchaeal species previously sequenced encodes multiple members of the family. *Hfx. volcanii* encodes five family CspA homologs - HVO_A0615, HVO_1233, HVO_0497, HVO_0498, and HVO_1992. One (HVO_0498) corresponds to a protein annotated as CMI9 (which stands for conditioned medium induced protein 9) in Genbank (Bitan-Banin,G. and Mevarech,M., GenBank AAL35837, unpublished.)

#### Signal transduction

*Hfx. volcanii* contains an extensive array of signal transduction systems that comprise 4% of its genome (details are available in the MiST database [136]. Most noticeably, the organism possesses 135 one-component systems, single protein molecules containing both sensory and regulatory domains [137] – more than any archaeal genome sequenced to date. One-component systems are the predominant mode of signal transduction in bacteria and especially in archaea [137]. Although the *Hfx. volcanii* genome is not the largest among archaea, it contains 164 DNA-binding regulatory domains (*e.g.*, helix-turn-helix), the highest number among all archaea suggesting transcriptional regulation is particularly important in this species signal transduction. Comparative analysis of Halobacteria including *Hfx. volcanii* indicates that this group has complex signal transduction pathways. The number of protein domains implicated in signal transduction, such as small-ligand binding, protein-protein interactions and chemotaxis, is significantly higher in Halobacteria than in other archaea [137]. Below we discuss two major categories of signal transduction related genes and processes: ArcR/IciR transcriptional regulators and chemotaxis and motility systems.

Twenty-two ArcR-type (IclR-domain containing) transcriptional regulators are encoded in the genome of *Hfx. volcanii*, seven times more than a median amount found in Halobacteria and twice that of the highest number reported in archaea to date. The ArcR regulator in *Halobacterium* sp. NRC-1 is a part of the *arcRACB* operon that encodes enzymes for fermentative growth via the arginine deiminase pathway [138]. An arginine-ornithine antiporter gene, *arcD*, is located immediately downstream of this operon and is also the part of this pathway [139]. A putative ArcR ortholog in *Hfx. volcanii* (HVO_2092, 45% identity) is encoded next to the *arcD* ortholog (HVO_2093, 52% identity), which is transcribed in the opposite direction, but there are no *arcACB* genes in the vicinity: *arcA* and *arcC* are missing from the genome and the *arcB* ortholog (HVO_0041, 46% identity) is encoded elsewhere. Thus, the ArcR ortholog and other members of this protein family are predicted to control functions other than the arginine deiminase pathway. Notably, three *arcR/iclR* genes are located in the regions transcribed under low salt (12%) conditions. This might indicate that at least some regulators of this type have been co-opted for new regulatory processes such as changes in salinity or high stress. Accordingly, twenty of these transcriptional regulators have the pI in the range from 4.3 to 6.3, indicating a possible flexible range of optimal functionality under varied salt concentrations.

*Hfx. volcanii* possess a complete set of chemotaxis genes [140], which is encoded in two distinct clusters. The first gene cluster contains the genes encoding the central regulator of chemotaxis CheA (HVO_1223), adaptor protein CheW (HVO_1225), methylesterase CheB (HVO_1224) and a CheR-like methyltransferase (HVO_1222). The second cluster is comprised of genes encoding the CheY response regulator (HVO_1207), phosphatase CheC (HVO_1206) and deamidase CheD (HVO_1205), which follow genes encoding flagellin and flagella biosynthesis proteins. Twelve chemoreceptor genes are scattered through the genome. They belong to the archael/firmicute class and have conserved methylation sites that are required for sensory adaptation [140]. The chemoreceptor family includes two homologs ((HVO_1126 and HVO_1484) of the HemAT aerotaxis sensor [141]. There is a gene pair, which is orthologous to the *basB/basT* pair in *Hbt. salinarum*, being involved in taxis towards branched-chain and sulfur-containing amino acids. The *basT* gene (HVO_0554) encodes a transducer while the protein encoded by the *basB* (HVO_0553) belongs to the ABC transporter substrate-binding protein superfamily. Thus, similarly to a model archaeon for motility and chemotaxis (*Hbt. salinarum*), *Hfx. volcanii* is predicted to be motile and chemotactic toward a variety of environmental signals including oxygen.

### Conclusions

Extensive genetic, molecular biological as well as biochemical analyses of a diverse set of *Hfx. volcanii* cellular processes has significantly advanced our understanding of the biology of this model organism as well as of haloarchaea and archaea in general. Analysis of the complete genome sequence of *Hfx. volcanii* described here not only expanded this knowledge but also significantly enhances the value of *Hfx. volcanii* as a model organism for ongoing studies of archaeal biology. As the vast diversity not only among archaea but even among haloarchaea become apparent, and as more and more genomes of these species become available [142], it is invaluable to have several archaea that are amenable to biochemical, genetic as well as various genome-wide experimental studies, as the comparison of the similarities and differences among these organisms will help us to understand archaeal biology in general.

## Materials and Methods

### Library construction/sequencing/closure

*Haloferax volcanii* strain DS2 was obtained from ATCC (ATCC 29605) and grown in the recommended media. DNA was isolated using a Qiagen kit and used for genome sequencing. The complete genome sequence was determined using the whole-genome shotgun method [143]. For the random shotgun-sequencing phase, libraries with average sizes of 1.5–2.0 kb and 4.0–8.0 kb were used.

### Assembly and closure of the genome

The shotgun sequence data were assembled using the TIGR assembler [144] and genome closure/finishing was performed using a combination of primer walking, PCR, and genomic DNA sequencing as in [145]. The final assembly was checked to ensure that every base was included in at least two clones and was sequenced at least once in each direction. The average depth of coverage for the genome was 8.68-fold. When experimental evidence suggested that an origin of replication sequence was absent in the assembly of pHV4, the assembly was manually examined. Mate-pair reads were checked for consistency. A search of sequences from non-assembled bins did not detect the proposed missing sequence; likewise, no discrepancies in coverage could be found for the region proposed to contain the missing sequences.

### CDS identification and functional prediction

The GLIMMER3 program, an updated version of the well-known GLIMMER2 [146] gene finder, was used to identify putative CDSs [147]. Advantages of GLIMMER3 include improved start-site prediction and an HMM-inspired algorithm that limits the prediction of overlapping ORFs to defined levels. Putative CDSs were discarded when they had no significant sequence similarity to known genes and also overlapped CDSs that had significant sequence similarity to known genes. The ORF set was manually curated, especially with respect to start codon selection, as described for *Nmn. pharaonis* [128] using similarity-based checking [148]. Start codon prediction for secreted proteins could be enhanced by searching for Tat-lipobox and prelipin peptidase (PibD)-cleavage motifs [22], [128], [131]. Non-coding RNAs were identified as described previously [149]. Gene function annotation was based on results of BLASTP searches against Genpept and all completed microbial genomes, and on hidden Markov model searches of the PFAM and TIGRFAM databases [150], [151]. The annotation was manually curated by the *Haloferax* community. GC skew and nucleotide composition analysis were performed as described previously [149].

### RNA gene prediction

Transfer RNA genes were identified with tRNAscan-SE version 1.23 using the archaeal search mode and default cutoffs [152]. Ribosomal RNAs were identified with BLASTN [152], [153] against the previously sequenced *Hfx. volcanii* rRNA sequences. C/D box RNAs were searched using a version of snoscan [154] optimized for archaeal sRNAs [155]. RNaseP and SRP RNAs were identified using a relaxed BLASTN search (Evalue = 0.1) against the Rfam database (Rel 8.0) [156], then searching all hits against respective Rfam models using cmsearch within the INFERNAL covariance model package (v.0.7) using the local alignment option for maximum sensitivity.

### pI predictions

Calculations of the predicted pI of individual proteins were made with the Bioperl module Bio::Tools::pICalculator using the EMBL matrix to calculate pKa.

### Codon usage

A SCU table was generated for each individual replicon. These were compared with SCU tables generated from a 30 ORF moving window along each replicon. χ^2^ analysis (40 degrees of freedom, Stop, Met and Trp codons were omitted) was carried out to determine whether the SCU of each window of ORFs was statistically similar to each whole-replicon SCU. Comparisons were carried out in an automated manner using a C++ encoded program, SCUBA (Synonymous Codon Usage Bias Analysis).

### Phylogenetic profiles

Phylogenetic profiles were constructed to compare the complete set of *Hfx. volcanii* CDSs to available complete bacterial and archaeal genomes. For each predicted protein in the *Hfx. volcanii* genome, a profile was constructed reflecting the presence or absence of a homolog in all other query genomes. The *Hfx. volcanii* proteome was searched against complete proteomes using the BLASTP algorithm and its phylogenetic profile was analyzed as previously described [157]. If BLASTP returned an e-value less than or equal to 1×10^−5^, a value of 1 was given to that sequence to indicate its presence in a given species. Conversely, if the e-value was greater than 1×10^−5^, a value of 0 was returned, indicating the absence of the protein in a proteome. These results for each protein in the proteome for each species were then hierarchically clustered and viewed using TreeView [158].

### Automated Phylogenetic Inference System (APIS)

APIS (Automated Phylogenetic Inference System) automatically creates and summarizes the phylogenetic tree for each protein encoded by a genome (Badger *et al.*, unpublished). It is implemented as a series of Ruby scripts; the data and results can be viewed in an interactive manner via a web server. The homologs assigned by APIS to each phylogenetic tree are obtained by using WU-BLAST to compare the query protein against a curated database of proteins from complete genomes [153]. The full-length sequences of these homologs are then retrieved from the database and aligned using MUSCLE [159]. Bootstrapped neighbor-joining trees are generated using QuickTree [160]. Since, unlike most similar programs, QuickTree produces bootstrapped trees with meaningful branch lengths, the inferred tree is midpoint rooted prior to analysis. This makes possible the automatic determination of the taxonomic classification of the organisms with proteins in the same clade as the query protein. APIS was created to address some of the weaknesses of existing automated phylogenetic systems, such as PyPhy [161]. The use of a general-purpose protein database (*e.g.*, Swiss Prot [162]) by those systems weakens the resultant interpretation of clades because the absence of proteins from organisms which have not had their genomes completely sequenced cannot be taken as biological evidence for the nonexistence of such proteins.

### Accession numbers

The sequence data and annotation is available in Genbank with the following identification numbers: CP001953 (pHV3), CP001954 (pHV2), CP001955 (pHV4), CP001956 (main chromosome), and CP001957 (pHV1). The data is also available in the UCSC Archaeal Genome Browser at http://archaea.ucsc.edu/cgi-bin/hgGateway?db=haloVolc1. A summary of “Minimum information about a genome sequence” (MIGS) for this organism is presented in Table 5.

**Table 5:** Classification and general features of *Hfx. volcanii* DS2 and this genome sequencing project according to the “Minimum information about a genome sequence (MIGS)” specifications [163].

Category	Detail	Evidence code 1
Display Name (*)	*Haloferax volcanii* DS2	TAS [2]
NCBI Taxon ID (*)	309800	
Domain (*)	Archaea	TAS [2]
Phylum (*)	Euryarchaeota	TAS [2]
Class (*)	Halomebacteria	TAS [2]
Order	Halobacteriales	TAS [2]
Family	Halobacteriaceae	TAS [2]
Genus (*)	*Haloferax*	TAS [2]
Species (*)	*volcanii*	TAS [2]
Strain (*)	DS2	TAS [45]
Culture Collection ID	ATCC 26905	
Biosafety Level	1	NTAS
**Project Information**		
Project description	The complete genome sequence of *Haloferax volcanii* DS2, a model archaeon	
Accession #s	Genbank CP001953 (pHV3), CP001954 (pHV2), CP001955 (pHV4), CP001956 (main chromosome), CP001957 (pHV1)	This study
Project Type (*)	Genome	This study
Project Status (*)	Complete and published	This study
Contact Name (*)	Jonathan Eisen	This study
Contact Email (*)	jaeisen@ucdavis.edu	This study
GC Percent	65	This study
Sequencing Center Name (*)	TIGR, now part of JCVI	This study
Sequencing Center url (*)	http://www.jcvi.org	This study
Funding Agency Name	National Science Foundation	This study
Funding Agency url	http://www.nsf.gov	This study
Publication Journal	PLoS One	This study
Publication Volume	TBD	This study
Publication link (url)	TBD	This study
**Sequencing Information**		
Sequencing Status (*)	Complete	This study
Comments on Sequencing	Whole genome shotgun sequencing and finishing with Sanger method	This study
Library Method	1.5–2 kb, 4–8 kb	This study
Vector	pHOS2	This study
Assembly Method	TIGR assembler + manual curation	This study
Sequencing Depth	8.68	This study
Gene Calling Method	GLIMMER3 + manual curation	This study
Sequencing Method	Sanger	This study
Contig Count	5	This study
Estimated Size (in Kb)	4012	This study
Chromosome Count	4	This study
Plasmid Count	1	This study
Sequencing Country	USA	This study
**Environmental Metadata**		
Isolation Site	Dead Sea	TAS [45]
Source of Isolate	Shore mud, Northern end	TAS [45]
Isolation Country	Israel	TAS [45]
Isolation Pubmed ID	1190944	NTAS
Altitude	−400 m	TAS [45]
Depth	1 m	TAS [45]
**Organism Metadata**		
Oxygen Requirement	Aerobe	TAS [45]
Cell Shape	Highly pleomorphic	TAS [45]
Motility	Motile	TAS^2^
Sporulation	Nonsporulating	NTAS
Temperature Range	30–40°C	TAS [45]
Temperature Optimum	45°C	TAS [164]
Salinity	Halophile	TAS [45]
Cell Diameter	0.4–0.5 um	TAS [45]
Cell Length	1–3 um	TAS [45]
Color	Pink	TAS [45]
Gram Staining	Negative	TAS [45]
Diseases	None	NTAS
Habitat	Hypersaline water	TAS [45]
Energy Sources	Carbohydrates	TAS [45]

1 Evidence codes - TAS: Traceable Author Statement (*i.e.*, a direct report exists in the literature); NAS: Non-traceable Author Statement (*i.e.*, not directly observed for the living, isolated sample, but based on a generally accepted property for the species, or anecdotal evidence).

2 *Hfx. volcanii*, which was long considered to be non-motile, has recently been shown to exhibit flagella-dependent motility (Pohlschroder, submitted), consistent with the presence of the *fla* genes in its genome.

## Supporting Information

Text S1 Text and references relating to Tables S1, S2, and S3 and genome mapping of historical experimental data.
(0.06 MB DOC)

Table S1 Superimposition of genetic map onto genome sequence. Table 3 from the original genetic map paper [12] was used to superimpose the map onto the genome sequence annotation. Median distance between map marker gene start and end and genome sequence marker gene start and end was calculated and was used to calculate a conversion factor between these two sequences. Coordinates of the annotated genome sequence are equivalent to 934.455 kb + x, where x is the position in the original genetic map. * indicates genes that showed a marked discrepancy in the length of the marker gene as determined by the genetic map, and were therefore removed from the median calculation. The mean of all start and end differences: 933455.5.
(0.07 MB DOC)

Table S2 Mapping transcription studies onto the genome sequence. Using the conversion factor described above (934.455+ x) the coordinates of transcription induction were taken from tables in the original studies by Ferrer et al. [8] and Trieselmann and Charlebois [9] and coordinated with the genome sequence annotation.
(0.04 MB DOC)

Table S3 Genes found in transcriptionally induced regions. The genome coordinates determined in Table S2 were used to extract the gene names and descriptions from the genome annotation in the context of the physiological description associated with these regions in the original studies. The regions of *Hfx. volcanii* genetic map were transcribed in response to low (12%) and high (30%) salt concentrations, different growth media, or heat shock [8], [9].
(0.79 MB DOC)
