# Supplementary material for: The Complete Genome Sequence of Haloferax volcanii DS2, a Model Archaeon
Source: PLoS One. 2010 Mar 19;5(3):e9605. doi: 10.1371/journal.pone.0009605 (PMC2841640; doi:10.1371/journal.pone.0009605)
Supplement: Text S1 — Text and references relating to Tables S1, S2, and S3 and genome mapping of historical experimental data. (0.06 MB DOC) [file pone.0009605.s001.doc]

The *Halobacteriaceae* differ in many ways from other non-archaeal halophiles and extreme halophiles. Most *Halobacteriaceae* are thought to be obligate halophiles as they cannot grow at salt concentrations below about 1M (although slow growth and survival has been reported at concentrations as low as 0.4 M [1]). Their strategy for tolerating high salt differs markedly from that used by the halophilic bacteria (with the exception of *Salinibacter ruber*) and by eukaryotes and allows them to withstand higher concentrations of salt.

Rather than accumulating organic solutes or continuously pumping salt out of the cell, the *Halobacteriaceae* equilibrate to high external salt concentrations by accumulating intracellular cations, or counterions, such as K^+^. As a result, haloarchaeal proteins possess a highly charged surface surrounded by a hydration shell, which prevents aggregation, thus enabling these proteins to function under high salt conditions.

When adapting to a low-salt environment the cell must efflux internal potassium ions; in contrast, if the external salt concentration increases, the cell must increase the internal potassium concentration. Therefore, low salt adaptation should be a slower, energy-consuming process. (For a review of these processes, see [2].)

For over a decade, *Hfx. volcanii* has been a model organism for the study of osmoregulation in high salt environments. Many of its responses to salt challenge have been characterized, including global changes in transcription patterns, the up-regulation of salt-induced proteins, the induction of other specific biochemical pathways, and changes in protein surfaces and DNA topology [3,4,5,6,7]. Previous studies identified regions of the original *Hfx. volcanii* genetic map that are transcribed in response to low (12%) or high (30%) salt concentrations [8,9].

When those regions were superimposed onto the genome sequence (Table S2 using conversion factor determined in Table S1), they were found to include genes known to be involved in osmotic homeostasis (Table S3). Genes of unknown function also mapped to these regions, suggesting that future studies might uncover new genes involved in osmotic adaptation. However, our results must be interpreted with some caution due to the low resolution of the original genetic map.

Most significantly, a clear “high salt adaptation region” was identified extending from position 956545 to 9875454 in the genome sequence, corresponding to clones 347 and 196 in the original genetic map (Table S3). This region harbors the only Na^+^/H^+^ antiporter and *trkA* potassium uptake system operons identified in the genome annotation, both of which are essential for haloarchaeal osmoregulation [2,10]. This finding provides a “proof-of-principle” that the original genetic map can, at least in part, be coordinated to the genome annotation.

In addition, a “stress response” region on the map that is transcribed in response to low salinity corresponded to position 1426545 to 1476545 in the genome sequence. This region contains *grpE*, *dnaK*, *dnaJ*, and other chaperones, as well as DNA repair genes including DNA gyrase and topoisomerase VI (*topoVI*), suggesting that this is a “damage response” region that can respond to either protein or DNA damage (Table S3).

A region associated with increased transcription at high salinity and decreased transcription at low salinity matched with genome positions 1964295 to 2014295. This region contains several complete sugar transporter complexes: a phosphotransferase system (PTS) operon, two complete ABC sugar transporters, and an oligopeptide ABC transporter (Table S3). It also encodes three ArcR regulators. Notably, the *arcR* gene family is expanded within the haloarchaea (see main text), thus creating the possibility of additional regulators for some of these genes.

In general, whereas the halophilic bacteria use organic solutes, such as sugars, for osmotic adaptation, haloarchaea are thought to rely on the well-studied mechanism of potassium accumulation. However, finding a sugar transport system in the putatively high salt-transcribed region could suggest a novel role for sugars in *Haloferax*’s osmoregulation processes. For example, in the bacterium *Listeria monocytogenes*, the PTS is known to be important for salt adaptation [11]. This could be a feature unique to *Hfx. volcanii* as it is the only haloarchaeon—or more broadly, the only archaeon—to possess multiple bacterial-like PTSs, including *both* a cytosolic PTS complex and complete membrane-bound enzyme II complexes.

Supporting Information References:

1. Soppa J (2005) From replication to cultivation: hot news from Haloarchaea. Curr Opin Microbiol 8: 737-744.

2. Oren A (2006) Life at high salt concentrations. In: Martin Dworkin SF, Eugene Rosenberg, Karl-Heinz Schleifer and Erko Stackebrandt, editor. Prokaryotes. 3rd ed: Springer New York. pp. 263-282.

3. Bidle KA, Hanson TE, Howell K, Nannen J (2006) HMG-CoA reductase is regulated by salinity at the level of transcription in *Haloferax* *volcanii*. Extremophiles.

4. Mojica FJ, Cisneros E, Ferrer C, Rodriguez-Valera F, Juez G (1997) Osmotically induced response in representatives of halophilic prokaryotes: the bacterium *Halomonas* *elongata* and the archaeon *Haloferax* *volcanii*. J Bacteriol 179: 5471-5481.

5. Bidle KA (2003) Differential expression of genes influenced by changing salinity using RNA arbitrarily primed PCR in the archaeal halophile *Haloferax* *volcanii*. Extremophiles 7: 1-7.

6. Madern D, Camacho M, Rodriguez-Arnedo A, Bonete MJ, Zaccai G (2004) Salt-dependent studies of NADP-dependent isocitrate dehydrogenase from the halophilic archaeon *Haloferax* *volcanii*. Extremophiles 8: 377-384.

7. Mojica FJ, Charbonnier F, Juez G, Rodriguez-Valera F, Forterre P (1994) Effects of salt and temperature on plasmid topology in the halophilic archaeon *Haloferax* *volcanii*. J Bacteriol 176: 4966-4973.

8. Ferrer C, Mojica FJ, Juez G, Rodriguez-Valera F (1996) Differentially transcribed regions of *Haloferax* *volcanii* genome depending on the medium salinity. J Bacteriol 178: 309-313.

9. Trieselmann BA, Charlebois RL (1992) Transcriptionally active regions in the genome of the archaebacterium *Haloferax* *volcanii*. J Bacteriol 174: 30-34.

10. Meury J, Kohiyama M (1989) Atp Is Required for K+ Active-Transport in the Archaebacterium *Haloferax* v*olcanii*. Archives of Microbiology 151: 530-536.

11. Duche O, Tremoulet F, Glaser P, Labadie J (2002) Salt stress proteins induced in *Listeria* *monocytogenes*. Appl Environ Microbiol 68: 1491-1498.

12. Charlebois RL, Schalkwyk LC, Hofman JD, Doolittle WF (1991) Detailed physical map and set of overlapping clones covering the genome of the archaebacterium *Haloferax* *volcanii* DS2. J Mol Biol 222: 509-524.
