# Supplementary material for: The Complete Genome Sequence of Haloferax volcanii DS2, a Model Archaeon
Source: PLoS One. 2010 Mar 19;5(3):e9605. doi: 10.1371/journal.pone.0009605 (PMC2841640; doi:10.1371/journal.pone.0009605)
Supplement: Table S1 — Superimposition of genetic map onto genome sequence. Table 3 from the original genetic map paper [12] was used to superimpose the map onto the genome sequence annotation. Median distance between map marker gene start and end and genome sequence marker gene start and end was calculated and was used to calculate a conversion factor between these two sequences. Coordinates of the annotated genome sequence are equivalent to 934.455 kb + x, where x is the position in the original genetic map. * indicates genes that showed a marked discrepancy in the length of the marker gene as determined by the genetic map, and were therefore removed from the median calculation. The mean of all start and end differences: 933455.5. (0.07 MB DOC) [file pone.0009605.s002.doc]

|  | **Chrom.Start** | **Chrom.End** | **Map Start** | **Map End** | **Start Diff.** | **End Diff.** |
| --- | --- | --- | --- | --- | --- | --- |
| Hvo-SSU-1 | 1598192 | 1599664 | 2557000 | 2559000 | 958808 | 959336 |
| Hvo-SSU-2 | 2770163 | 2771635 | 854000 | 855000 | 931594 | 931122 |
| Hvo-LSU-1 | 1600047 | 1602961 | 2559000 | 2562000 | 958953 | 959039 |
| Hvo-LSU-2 | 2766866 | 2769780 | 850000 | 853000 | 930891 | 930977 |
| Hvo-5S-1 | 1603075 | 1603196 | 2562000 | 2562150 | 958925 | 958954 |
| Hvo-5S-2 | 2766631 | 2766752 | 850000 | 850150 | 931126 | 931155 |
| Hvo-*RNaseP* | 1664260 | 1665405 | 2611000 | 2645000 | 946740 | *979595 |
| *csg* | 1932444 | 1932884 | 8000 | 11000 | 923313 | 925873 |
| *folA* | 1166683 | 1167171 | 2102000 | 2109000 | 935317 | 941829 |
| *gyrB* | 1441393 | 1443312 | 2394000 | 2404000 | 952607 | 960688 |
| *hisC* | 1179103 | 1180188 | 2116000 | 2117000 | 936897 | 936812 |
| *hmg* | 2434849 | 2436060 | 507000 | 511000 | 919908 | 922697 |
| *rplL* | 2594860 | 2595201 | 684000 | 700000 | 936897 | 952556 |
| *sodC* | 2749866 | 2750465 | 819000 | 826000 | 916891 | 923292 |
| *trpA* | 708729 | 709562 | 1635000 | 1636000 | 926271 | 926438 |
| *trpB* | 707461 | 708729 | 1633000 | 1635000 | 925539 | 926271 |
