# Supplementary material for: The Complete Genome Sequence of Haloferax volcanii DS2, a Model Archaeon
Source: PLoS One. 2010 Mar 19;5(3):e9605. doi: 10.1371/journal.pone.0009605 (PMC2841640; doi:10.1371/journal.pone.0009605)
Supplement: Table S2 — Mapping transcription studies onto the genome sequence. Using the conversion factor described above (934.455+ x) the coordinates of transcription induction were taken from tables in the original studies by Ferrer et al. [8] and Trieselmann and Charlebois [9] and coordinated with the genome sequence annotation. (0.04 MB DOC) [file pone.0009605.s003.doc]

| **Map Cosmid** | **Map start (kb)** | **Map end (kb)** | **Genome start** | **Genome end** |
| --- | --- | --- | --- | --- |
| 261 | 50 | 100 | 1964295 | 2014295 |
| 531 | 450 | 460 | 2364295 | 2374295 |
| 266 | 460 | 480 | 2374295 | 2394295 |
| 196 | 1890 | 1921 | 956545 | 987545 |
| 5G7 | 2360 | 2410 | 1426545 | 1476545 |
| 41 | 2660 | 2700 | 1726545 | 1766545 |
| 437 | 2690 | 2840 | 1756545 | 1906545 |
| RE-416 | 348 | 365 | 2262295 | 2279295 |
| CE-531/D57 | 459 | 464 | 2373295 | 2378295 |
| HS-531/D57 | 459 | 464 | 2373295 | 2378295 |
| HS-H11 | 624 | 644 | 2538295 | 2558295 |
| HS-A199 | 1037 | 1058 | 103545 | 124545 |
| HS-268 | 1318 | 1333 | 384545 | 399545 |
| HS-456/A210 | 1370 | 1390 | 436545 | 456545 |
| HS-452 | 1616 | 1638 | 682545 | 704545 |
| HS-10D2/470 | 2799 | 2819 | 1865545 | 1885545 |
