# Supplementary material for: The Complete Genome Sequence of Haloferax volcanii DS2, a Model Archaeon
Source: PLoS One. 2010 Mar 19;5(3):e9605. doi: 10.1371/journal.pone.0009605 (PMC2841640; doi:10.1371/journal.pone.0009605)
Supplement: Table S3 — Genes found in transcriptionally induced regions. The genome coordinates determined in Table S2 were used to extract the gene names and descriptions from the genome annotation in the context of the physiological description associated with these regions in the original studies. The regions of Hfx. volcanii genetic map were transcribed in response to low (12%) and high (30%) salt concentrations, different growth media, or heat shock [8], [9]. (0.79 MB DOC) [file pone.0009605.s004.doc]

| **Map start** | **Map end** | **Map Clone** | **CDS** | **Chr. Start** | **Chr. End** | **Description/Comment** |
| --- | --- | --- | --- | --- | --- | --- |
| **1037** | **1058** | **A199** |  | **91500** | **112500** | **Heat shock region** |
|  |  |  | HVO_0093 | 90921 | 91928 | conserved hypothetical protein |
|  |  |  | HVO_0094 | 92064 | 92270 | conserved hypothetical protein |
|  |  |  | HVO_0095 | 92298 | 92993 | conserved hypothetical protein |
|  |  |  | HVO_0096 | 93097 | 94029 | dtdp glucose46 dehydratase |
|  |  |  | HVO_0097 | 94154 | 95038 | Uncharacterized protein family (UPF0153) family |
|  |  |  | HVO_0098 | 95156 | 95359 | hypothetical protein |
|  |  |  | HVO_0099 | 95441 | 97351 | conserved hypothetical protein |
|  |  |  | HVO_0099_A | 97756 | 97950 | conserved hypothetical protein |
|  |  |  | HVO_0100 | 98087 | 99214 | putative nonsense mediated mRNA decay protein |
|  |  |  | HVO_0101 | 99292 | 99930 | phosphoribosyltransferase |
|  |  |  | HVO_0102 | 100055 | 100936 | htpX HtpX protease homolog transmembrane |
|  |  |  | HVO_0103 | 100943 | 101548 | conserved hypothetical protein |
|  |  |  | HVO_0104 | 101807 | 102838 | radA DNA repair and recombination protein RadA |
|  |  |  | HVO_0105 | 103089 | 104234 | flavoprotein reductase homolog |
|  |  |  | HVO_0106 | 104245 | 104658 | conserved hypothetical protein |
|  |  |  | HVO_0107 | 104705 | 105133 | sufA FeS assembly protein SufA |
|  |  |  | HVO_0108 | 105206 | 105793 | conserved hypothetical protein |
|  |  |  | HVO_0109 | 105867 | 107141 | sufS cysteine desulfurase |
|  |  |  | HVO_0110 | 107327 | 108373 | HTRlike protein |
|  |  |  | HVO_0111 | 108419 | 108721 | Protein of unknown function (DUF424) superfamily |
|  |  |  | HVO_0112 | 108722 | 109462 | tetratricopeptide repeat protein |
|  |  |  | HVO_0113 | 109554 | 110171 | phosphoglycerate mutase family protein possible fructose2 6bisphosphatase |
|  |  |  | HVO_0114 | 110198 | 110752 | 2'5' RNA ligase |
|  |  |  | HVO_0115 | 110903 | 111055 | ribosomal protein L39.eR |
|  |  |  | HVO_0116 | 111059 | 111337 | ribosomal protein L31.eR |
|  |  |  | HVO_0117 | 111341 | 112006 | ribosome anti-association protein (translation initiation factor aIF-6) |
|  |  |  | HVO_0118 | 112088 | 112264 | ribosomal protein HL32 (LX) |
|  |  |  | HVO_0119 | 112261 | 112734 | pfdA prefoldin alpha subunit |
| **1318** | **1333** | **268** |  | **372500** | **387500** | **Heat shock region** |
|  |  |  | HVO_0417 | 371518 | 373026 | cxp metaldependent carboxypeptidase |
|  |  |  | HVO_0418 | 373221 | 374717 | HNH endonuclease domain protein |
|  |  |  | HVO_0419 | 374824 | 375918 | acetylornithine deacetylase |
|  |  |  | HVO_0420 | 376085 | 377716 | mpcT transducer protein MpcT |
|  |  |  | HVO_0421 | 377834 | 379714 | Ploop ATPase of the PilT family |
|  |  |  | HVO_0422 | 379829 | 380122 | conserved hypothetical protein |
|  |  |  | HVO_0423 | 380247 | 380561 | conserved hypothetical protein |
|  |  |  | HVO_0424 | 380564 | 382378 | ABC-type transport system ATP-binding protein |
|  |  |  | HVO_0425 | 382515 | 383456 | Glyoxalase I YfiE |
|  |  |  | HVO_0426 | 383511 | 383993 | conserved hypothetical protein TIGR00481 |
|  |  |  | HVO_0427 | 384018 | 385076 | rtcA RNA 3'terminal phosphate cyclase |
|  |  |  | HVO_0428 | 385077 | 385526 | uspA domain protein |
|  |  |  | HVO_0429 | 385608 | 386129 | putative metalloprotease DUF1695 |
|  |  |  | HVO_0430 | 386169 | 386621 | Hypothetical UPF0146 protein Vng2609c. |
|  |  |  | HVO_0431 | 386653 | 387507 | HAD superfamily (subfamily IA) hydrolase |
| **1370** | **1390** | **456/A210** |  | **424500** | **444500** | **Heat shock region** |
|  |  |  | HVO_0475 | 423626 | 424564 | hypothetical protein |
|  |  |  | HVO_0476 | 424661 | 425317 | conserved protein |
|  |  |  | HVO_0477 | 425456 | 426415 | ampS1 leucyl aminopeptidase |
|  |  |  | HVO_0478 | 426605 | 427672 | glyceraldehyde3phosphate dehydrogenase type II |
|  |  |  | HVO_0479 | 427742 | 428230 | conserved protein |
|  |  |  | HVO_0480 | 428646 | 429851 | pgk phosphoglycerate kinase |
|  |  |  | HVO_0481 | 429927 | 430979 | gap glyceraldehyde3phosphate dehydrogenase type I |
|  |  |  | HVO_0482 | 431247 | 431609 | hsp20-type molecular chaperone |
|  |  |  | HVO_0483 | 431634 | 432506 | rimK2 rimK family protein |
|  |  |  | HVO_0484 | 432592 | 433122 | ribosomal protein L10.eR |
|  |  |  | HVO_0485 | 433283 | 433930 | GMP synthase |
|  |  |  | HVO_0486 | 433931 | 434725 | nucS recB family nuclease |
|  |  |  | HVO_0487 | 434848 | 435303 | Pyridoxamine 5'phosphate oxidase family |
|  |  |  | HVO_0488 | 435432 | 435830 | conserved hypothetical protein |
|  |  |  | HVO_0489 | 435901 | 436059 | conserved hypothetical protein |
|  |  |  | HVO_0490 | 436184 | 436330 | conserved hypothetical protein |
|  |  |  | HVO_0491 | 436448 | 436909 | Pyridoxamine 5'phosphate oxidase family |
|  |  |  | HVO_0492 | 437012 | 437659 | boa bacterioopsin activatorlike protein |
|  |  |  | HVO_0493 | 437774 | 438166 | conserved hypothetical protein |
|  |  |  | HVO_0494 | 438284 | 439078 | conserved hypothetical protein |
|  |  |  | HVO_0495 | 439239 | 439312 | tRNAPhe1 |
|  |  |  | HVO_0496 | 439362 | 440087 | conserved hypothetical protein |
|  |  |  | HVO_0497 | 440257 | 440451 | cspD1 cold shock protein |
|  |  |  | HVO_0498 | 440611 | 440805 | cspD2,cmi9 cold shock protein |
|  |  |  | HVO_0499 | 441236 | 441309 | tRNAPhe2 |
|  |  |  | HVO_0500 | 441371 | 442039 | conserved hypothetical protein |
|  |  |  | HVO_0501 | 442085 | 442483 | conserved hypothetical protein |
|  |  |  | HVO_0502 | 442587 | 442790 | hypothetical protein |
|  |  |  | HVO_0503 | 443018 | 443091 | tRNAVal1 |
|  |  |  | HVO_0504 | 443185 | 443682 | Uncharacterized ACR COG1430 |
|  |  |  | HVO_0505 | 443723 | 443992 | Putative dehydrogenase |
|  |  |  | HVO_0506 | 444097 | 446034 | ABC-type transport system ATP-binding/permease protein |
| **1616** | **1638** | **452** |  | **670500** | **692500** | **Heat shock region** |
|  |  |  | HVO_0749 | 670267 | 672030 | pilC2 type IV pilus biogenesis complex membrane subunit |
|  |  |  | HVO_0750 | 672085 | 672555 | conserved hypothetical protein |
|  |  |  | HVO_0751 | 672533 | 672925 | conserved hypothetical protein |
|  |  |  | HVO_0752 | 672922 | 673806 | conserved hypothetical protein |
|  |  |  | HVO_0753 | 673803 | 676859 | conserved hypothetical protein |
|  |  |  | HVO_0754 | 676918 | 677337 | conserved hypothetical protein |
|  |  |  | HVO_0755 | 677334 | 678230 | 3beta hydroxysteroid dehydrogenase/isomerase family superfamily |
|  |  |  | HVO_0756 | 678332 | 679516 | phosphoesterase RecJ domain protein |
|  |  |  | HVO_0757 | 679608 | 680699 | aldo/keto reductase |
|  |  |  | HVO_0758 | 680745 | 680915 | conserved hypothetical protein |
|  |  |  | HVO_0759 | 681073 | 682233 | sugar transporter |
|  |  |  | HVO_0760 | 682316 | 683422 | wcaA glycosyltransferase homolog putative (TBD) |
|  |  |  | HVO_0761 | 683419 | 683907 | conserved hypothetical protein |
|  |  |  | HVO_0762 | 683937 | 684533 | Mut/nudix family protein |
|  |  |  | HVO_0763 | 684671 | 685198 | terminal quinol oxidase |
|  |  |  | HVO_0764 | 685589 | 686371 | conserved hypothetical protein |
|  |  |  | HVO_0765 | 686654 | 687820 | soxB1 sarcosine oxidase |
|  |  |  | HVO_0766 | 687866 | 688354 | hsp20D hsp20-type molecular chaperone |
|  |  |  | HVO_0767 | 688351 | 688509 | conserved hypothetical protein |
|  |  |  | HVO_0768 | 688631 | 690328 | radical HhH |
|  |  |  | HVO_0769 | 690496 | 690900 | hypothetical protein (TBD) |
|  |  |  | HVO_0770 | 690965 | 691660 | conserved protein |
|  |  |  | HVO_0771 | 691757 | 692545 | probable metallo-beta-lactamase family hydrolase |
| **1890** | **1921** | **196** |  | **944500** | **975500** | **Alternating regions transcribed at low (12%) and high (30%) salt conditions** |
|  |  |  | HVO_1034 | 943502 | 945163 | pilB3 type IV pilus biogenesis complex ATPase subunit |
|  |  |  | HVO_1035 | 945296 | 945793 | conserved hypothetical protein |
|  |  |  | HVO_1036 | 945888 | 946256 | dsbh domain containing protein |
|  |  |  | HVO_1037 | 946398 | 946871 | conserved hypothetical protein |
|  |  |  | HVO_1038 | 947001 | 947627 | ugd4 uracil DNA glycosylase |
|  |  |  | HVO_1039 | 948031 | 948534 | metallobetalactamase superfamily protein |
|  |  |  | HVO_1040 | 948679 | 949818 | DnaJ domain protein |
|  |  |  | HVO_1041 | 949985 | 950983 | metallobetalactamase superfamily domain protein |
|  |  |  | HVO_1042 | 951010 | 951300 | rpoL DNA-directed RNA polymerase subunit L |
|  |  |  | HVO_1043 | 951364 | 951924 | Protein of unknown function (DUF1684) superfamily |
|  |  |  | HVO_1044 | 952064 | 952876 | hisF imidazoleglycerol phosphate synthase cyclase subunit |
|  |  |  | HVO_1045 | 953100 | 953222 | hypothetical protein |
|  |  |  | HVO_1046 | 953332 | 953499 | hypothetical protein |
|  |  |  | HVO_1047 | 953595 | 954566 | qor2 NADPH:quinone reductase |
|  |  |  | HVO_1048 | 954615 | 956723 | purL phosphoribosylformylglycinamidine synthase II |
|  |  |  | HVO_1049 | 956966 | 957649 | PHP domain protein |
|  |  |  | HVO_1050 | 957649 | 958740 | asnA asparagine synthetase |
|  |  |  | HVO_1051 | 958762 | 959244 | nudH Mut/nudix family protein |
|  |  |  | HVO_1052 | 959346 | 960344 | tfb1 transcription initiation factor TFB |
|  |  |  | HVO_1053 | 960564 | 960842 | aatC aspartyl-tRNA(Asn) amidotransferase subunit C |
|  |  |  | HVO_1054 | 960843 | 962114 | aatA aspartyl-tRNA(Asn) amidotransferase subunit A |
|  |  |  | HVO_1055 | 962244 | 962924 | trkA1 Trk potassium uptake system protein TrkA |
|  |  |  | HVO_1056 | 962924 | 964585 | trkH1 Trk potassium uptake system protein TrkH |
|  |  |  | HVO_1057 | 964613 | 966217 | trkH2 Trk potassium uptake system protein TrkH |
|  |  |  | HVO_1058 | 966335 | 967672 | trkA2 potassium uptake system protein TrkA |
|  |  |  | HVO_1059 | 967758 | 968300 | conserved hypothetical protein |
|  |  |  | HVO_1060 | 968419 | 969378 | conserved hypothetical protein |
|  |  |  | HVO_1061 | 969459 | 970238 | txrB thioredoxin reductase |
|  |  |  | HVO_1062 | 970333 | 971493 | coaBC phosphopantothenoylcysteine decarboxylase/phosphopantothenatecysteine ligase |
|  |  |  | HVO_1063 | 971509 | 971904 | Na(+) H(+) antiporter |
|  |  |  | HVO_1064 | 971901 | 972194 | Na(+)/H(+) antiporter subunit F |
|  |  |  | HVO_1065 | 972187 | 972789 | mrpE multiple resistance/pH regulation related protein E |
|  |  |  | HVO_1066 | 972789 | 974486 | NADHubiquinone/plastoquinone family protein |
|  |  |  | HVO_1067 | 974483 | 974824 | Na(+)/H(+) antiporter subunit C |
|  |  |  | HVO_1068 | 974821 | 975294 | phaA2 Multisubunit Na+/H+ antiporter MnhA and MnhB subunits |
|  |  |  | HVO_1069 | 975291 | 977696 | mrpA Na(+)/H(+) antiporter subunit A |
| **2360** | **2395** | **5G7 (307)** |  | **1414500** | **1449500** | **Strongly induced by low (12%) salinity** |
|  |  |  | HVO_1546 | 1414133 | 1415131 | dhaK dihydroxyacetone kinase dhaK subunit |
|  |  |  | HVO_1547 | 1415297 | 1418428 | ileS isoleucyltRNA synthetase |
|  |  |  | HVO_1548 | 1418809 | 1419417 | lipoprotein putative |
|  |  |  | HVO_1549 | 1419475 | 1419930 | conserved hypothetical protein |
|  |  |  | HVO_1550 | 1419994 | 1420476 | act2 acyl-CoA thioester hydrolase |
|  |  |  | HVO_1551 | 1420537 | 1420965 | Uncharacterized ACR COG1259 superfamily |
|  |  |  | HVO_1552 | 1421062 | 1421682 | putative bacterial regulatory protein arsR family |
|  |  |  | HVO_1553 | 1421746 | 1423074 | ttuD putative hydroxypyruvate reductase |
|  |  |  | HVO_1554 | 1423128 | 1424813 | traB TraB family protein |
|  |  |  | HVO_1555 | 1424810 | 1425433 | SpoIVFBtype metallopeptidase transmembrane (TBD) |
|  |  |  | HVO_1556 | 1425648 | 1425884 | hypothetical protein |
|  |  |  | HVO_1557 | 1426301 | 1427275 | purM phosphoribosylformylglycinamidine cycloligase |
|  |  |  | HVO_1558 | 1427348 | 1428652 | cytochrome P450 |
|  |  |  | HVO_1559 | 1428701 | 1429240 | HTH/CBS domain protein |
|  |  |  | HVO_1560 | 1429308 | 1429667 | Uncharacterized protein conserved in archaea |
|  |  |  | HVO_1561 | 1429956 | 1430198 | hypothetical protein |
|  |  |  | HVO_1562 | 1430347 | 1431078 | psmB proteasome beta subunit |
|  |  |  | HVO_1563 | 1431219 | 1431596 | hypothetical protein |
|  |  |  | HVO_1564 | 1431708 | 1433237 | conserved hypothetical protein |
|  |  |  | HVO_1565 | 1433356 | 1435113 | ligA DNA ligase ATP dependent |
|  |  |  | HVO_1566 | 1435259 | 1435537 | hypothetical protein |
|  |  |  | HVO_1567 | 1435666 | 1436373 | conserved hypothetical protein |
|  |  |  | HVO_1568 | 1436475 | 1437308 | HydD putative |
|  |  |  | HVO_1569 | 1437398 | 1437616 | hypothetical protein |
|  |  |  | HVO_1570 | 1437643 | 1438743 | top6A DNA topoisomerase VI subunit A |
|  |  |  | HVO_1571 | 1438743 | 1441139 | top6B DNA topoisomerase VI subunit B |
|  |  |  | HVO_1572 | 1441393 | 1443312 | gyrB DNA gyrase subunit B |
|  |  |  | HVO_1573 | 1443314 | 1445902 | gyrA DNA gyrase subunit A |
|  |  |  | HVO_1574 | 1446425 | 1446895 | MutT/nudix family protein |
|  |  |  | HVO_1575 | 1446895 | 1447794 | rocF arginase |
|  |  |  | HVO_1576 | 1447899 | 1448819 | galE3 UDPglucose 4epimerase |
| **2660** | **2700** | **41** |  | **1714500** | **1754500** | **Transcription enhancement at both high and low salinities** |
|  |  |  | HVO_1855 | 1714136 | 1715041 | Integral membrane protein DUF6 domain protein |
|  |  |  | HVO_1856 | 1715144 | 1715734 | predicted ATPase PPloop superfamily |
|  |  |  | HVO_1857 | 1715738 | 1716100 | conserved protein |
|  |  |  | HVO_1858 | 1716206 | 1716661 | rps19R ribosomal protein S19.eR |
|  |  |  | HVO_1859 | 1716776 | 1717804 | membrane protein putative |
|  |  |  | HVO_1860 | 1717837 | 1717962 | hypothetical protein |
|  |  |  | HVO_1861 | 1717971 | 1718864 | thiL thiaminemonophosphate kinase |
|  |  |  | HVO_1862 | 1718904 | 1720043 | S2P family metalloprotease transmembrane |
|  |  |  | HVO_1863 | 1720493 | 1720738 | conserved hypothetical protein |
|  |  |  | HVO_1864 | 1721045 | 1721830 | moaE molybdenum cofactor biosynthesis protein |
|  |  |  | HVO_1865 | 1721855 | 1722199 | hypothetical protein |
|  |  |  | HVO_1866 | 1722293 | 1723006 | uridylate kinase putative |
|  |  |  | HVO_1867 | 1723008 | 1724675 | lysS lysyltRNA synthetase |
|  |  |  | HVO_1868 | 1724727 | 1725266 | conserved hypothetical protein |
|  |  |  | HVO_1869 | 1725397 | 1725873 | hypothetical protein |
|  |  |  | HVO_1870 | 1726022 | 1727809 | S2P family metalloprotease transmembrane |
|  |  |  | HVO_1871 | 1727916 | 1729421 | pitA chlorite dismutase family protein |
|  |  |  | HVO_1872 | 1729602 | 1729928 | hypothetical protein |
|  |  |  | HVO_1873 | 1730131 | 1730301 | hypothetical protein |
|  |  |  | HVO_1874 | 1730301 | 1731374 | probable oxidoreductase (aldo-keto reductase family protein) |
|  |  |  | HVO_1875 | 1731564 | 1732481 | acetyltransferaselike |
|  |  |  | HVO_1876 | 1732593 | 1732892 | conserved hypothetical protein |
|  |  |  | HVO_1877 | 1733316 | 1733386 | tRNAGly2 |
|  |  |  | HVO_1878 | 1733825 | 1734655 | nadE NAD+ synthetase |
|  |  |  | HVO_1879 | 1734697 | 1735392 | fadA3 enoylCoA hydratase |
|  |  |  | HVO_1880 | 1735453 | 1736130 | conserved hypothetical protein |
|  |  |  | HVO_1881 | 1736209 | 1736280 | tRNAThr2 |
|  |  |  | HVO_1882 | 1736369 | 1737445 | pectin methylesterase |
|  |  |  | HVO_1883 | 1737687 | 1738298 | conserved hypothetical protein |
|  |  |  | HVO_1884 | 1738397 | 1739014 | conserved hypothetical protein |
|  |  |  | HVO_1885 | 1739055 | 1739708 | trkA3 Trk potassium uptake system protein |
|  |  |  | HVO_1886 | 1740018 | 1740884 | ABC-type transport system ATP-binding protein (probable substrate sulfate/tungstate) |
|  |  |  | HVO_1887 | 1740881 | 1741579 | ABC-type transport system permease protein (probable substrate sulfate/tungstate) |
|  |  |  | HVO_1888 | 1741604 | 1742632 | ABC-type transport system periplasmic substrate-binding protein (probable substrate sulfate/tungstate) |
|  |  |  | HVO_1889 | 1742864 | 1743340 | conserved hypothetical protein |
|  |  |  | HVO_1890 | 1743400 | 1743573 | hypothetical protein |
|  |  |  | HVO_1891 | 1743570 | 1743749 | hypothetical protein |
|  |  |  | HVO_1892 | 1743807 | 1744214 | conserved hypothetical protein |
|  |  |  | HVO_1893 | 1744375 | 1745007 | Ham1 family |
| **2690** | **2840** | **437 (470)** |  | **1744500** | **1894500** | **Transcription at high salinity** |
|  |  |  | HVO_1894 | 1745062 | 1745340 | conserved hypothetical protein |
|  |  |  | HVO_1895 | 1745405 | 1747063 | Putative KEOPS component Kae1-Bud32 |
|  |  |  | HVO_1896 | 1747097 | 1747402 | rps24R ribosomal protein S24.eR |
|  |  |  | HVO_1897 | 1747471 | 1748010 | conserved protein |
|  |  |  | HVO_1898 | 1748024 | 1748221 | rpoE2 DNAdirected RNA polymerase subunit E |
|  |  |  | HVO_1899 | 1748221 | 1748793 | rpoE1 DNAdirected RNA polymerase subunit E’ |
|  |  |  | HVO_1900 | 1748801 | 1749190 | Predicted RNAbinding protein containing PIN domain |
|  |  |  | HVO_1901 | 1749192 | 1750427 | translation initiation factor aIF-2 gamma subunit |
|  |  |  | HVO_1902 | 1750666 | 1751373 | conserved hypothetical protein |
|  |  |  | HVO_1903 | 1751599 | 1752582 | conserved hypothetical protein |
|  |  |  | HVO_1904 | 1752587 | 1752799 | hypothetical protein |
|  |  |  | HVO_1905 | 1752796 | 1753620 | phnP ATPbinding protein |
|  |  |  | HVO_1906 | 1754022 | 1754762 | hypothetical protein |
|  |  |  | HVO_1907 | 1754807 | 1756231 | cdc48d cell division control protein 48 |
|  |  |  | HVO_1908 | 1756594 | 1758696 | napA nitrate reductase |
|  |  |  | HVO_1909 | 1758755 | 1759386 | mobA3 molybdopterin-guanine dinucleotide biosynthesis protein A (nonfunctional) |
|  |  |  | HVO_1911 | 1759649 | 1761402 | nirA2 ferredoxin--nitrite reductase (nonfunctional) |
|  |  |  | HVO_1913 | 1761405 | 1761608 | hypothetical protein |
|  |  |  | HVO_1914 | 1761736 | 1762869 | 3ketoacylCoA thiolase |
|  |  |  | HVO_1915 | 1762997 | 1763173 | hypothetical protein |
|  |  |  | HVO_1916 | 1763225 | 1765132 | TrkAN domain family |
|  |  |  | HVO_1917 | 1765262 | 1767262 | acs6 acyl-CoA synthetase |
|  |  |  | HVO_1918 | 1767360 | 1768268 | metallobetalactamase superfamily domain protein |
|  |  |  | HVO_1919 | 1768410 | 1768697 | hypothetical protein |
|  |  |  | HVO_1920 | 1768729 | 1770297 | trnS sodium dependent transporter |
|  |  |  | HVO_1921 | 1770475 | 1771857 | serS seryltRNA synthetase |
|  |  |  | HVO_1922 | 1771907 | 1772260 | conserved hypothetical protein |
|  |  |  | HVO_1923 | 1772389 | 1772886 | Uncharacterized conserved protein |
|  |  |  | HVO_1924 | 1772932 | 1773294 | hypothetical protein. |
|  |  |  | HVO_1925 | 1773508 | 1774485 | gbp GTPbinding protein |
|  |  |  | HVO_1926 | 1774629 | 1775381 | Protein of unknown function (DUF541) superfamily |
|  |  |  | HVO_1927 | 1775472 | 1776776 | membrane protein putative |
|  |  |  | HVO_1928 | 1776819 | 1777550 | 5formyltetrahydrofolate cycloligase |
|  |  |  | HVO_1929 | 1777642 | 1778232 | putative DNA binding domain |
|  |  |  | HVO_1930_A | 1778718 | 1779050 | conserved hypothetical protein |
|  |  |  | HVO_1931 | 1779189 | 1779806 | GTPbinding protein |
|  |  |  | HVO_1932 | 1779866 | 1780792 | ddh 2-D-hydroxyacid dehydrogenase |
|  |  |  | HVO_1933 | 1780813 | 1781058 | conserved hypothetical protein |
|  |  |  | HVO_1934 | 1781433 | 1782674 | translation initiation factor aIF-2B alpha subunit |
|  |  |  | HVO_1935 | 1782735 | 1783229 | putative phosphoesterase |
|  |  |  | HVO_1936 | 1783347 | 1784102 | cofE F420-0:gamma-glutamyl ligase |
|  |  |  | HVO_1937 | 1784155 | 1785141 | Coenzyme F420-dependent N(5) N(10)methylenetetrahydromethanopterin reductase |
|  |  |  | HVO_1938 | 1785485 | 1785712 | conserved hypothetical protein |
|  |  |  | HVO_1939 | 1785835 | 1787526 | mutLa DNA mismatch repair protein MutL |
|  |  |  | HVO_1940 | 1787523 | 1790288 | mutS1a DNA mismatch repair protein MutS |
|  |  |  | HVO_1941 | 1790495 | 1791451 | methanol dehydrogenase regulatory protein |
|  |  |  | HVO_1942 | 1791457 | 1792485 | conserved hypothetical protein |
|  |  |  | HVO_1943 | 1792482 | 1794722 | Transglutaminaselike superfamily domain protein |
|  |  |  | HVO_1944 | 1794784 | 1796034 | conserved hypothetical protein |
|  |  |  | HVO_1945 | 1796200 | 1797279 | hypothetical protein |
|  |  |  | HVO_1946 | 1797346 | 1797639 | translation initiation factor aIF-1 |
|  |  |  | HVO_1947 | 1797762 | 1798127 | rhodaneselike domain protein |
|  |  |  | HVO_1948 | 1798128 | 1798691 | Mut/nudix family protein |
|  |  |  | HVO_1949 | 1798688 | 1799209 | conserved hypothetical protein |
|  |  |  | HVO_1950 | 1799234 | 1799752 | conserved hypothetical protein |
|  |  |  | HVO_1951 | 1800019 | 1800351 | conserved hypothetical protein |
|  |  |  | HVO_1952 | 1800526 | 1801410 | bacterioopsin activatorlike protein putative |
|  |  |  | HVO_1953 | 1801414 | 1803735 | 11domain light and oxygen sensing his kinase putative |
|  |  |  | HVO_1954 | 1803818 | 1804306 | rimI ribosomalproteinalanine acetyltransferase |
|  |  |  | HVO_1955 | 1804460 | 1806424 | citB1 aconitate hydratase |
|  |  |  | HVO_1956 | 1806697 | 1807170 | Probable deoxycytidine triphosphate deaminase |
|  |  |  | HVO_1957 | 1807573 | 1808811 | panB proteasomeactivating nucleotidase B |
|  |  |  | HVO_1958 | 1808923 | 1809396 | pyruvoyldependent arginine decarboxylase |
|  |  |  | HVO_1959 | 1809487 | 1810332 | signaltransducing histidine kinaselike |
|  |  |  | HVO_1960 | 1810464 | 1810847 | conserved hypothetical protein |
|  |  |  | HVO_1961 | 1811021 | 1811182 | conserved hypothetical protein |
|  |  |  | HVO_1962 | 1811208 | 1811531 | hypothetical protein |
|  |  |  | HVO_1963 | 1811649 | 1813451 | tif5B translation initiation factor aIF-5B |
|  |  |  | HVO_1964 | 1813650 | 1813895 | PRCbarrel domain putative |
|  |  |  | HVO_1965 | 1813914 | 1814372 | Nob1p homolog (TBD) |
|  |  |  | HVO_1966 | 1814406 | 1815143 | CAAX amino terminal protease family transmembrane |
|  |  |  | HVO_1967 | 1815330 | 1816622 | pgi glucose6phosphate isomerase |
|  |  |  | HVO_1968 | 1816695 | 1817228 | conserved hypothetical protein |
|  |  |  | HVO_1969 | 1817248 | 1817931 | DedA family family |
|  |  |  | HVO_1970 | 1817928 | 1818632 | PHP domain protein |
|  |  |  | HVO_1971 | 1818629 | 1819327 | Phosphatidylserine synthase putative |
|  |  |  | HVO_1972 | 1819455 | 1820090 | acetyltransferase GNAT family family |
|  |  |  | HVO_1973 | 1820205 | 1820708 | conserved hypothetical protein |
|  |  |  | HVO_1974 | 1820758 | 1821099 | conserved hypothetical protein |
|  |  |  | HVO_1975 | 1821227 | 1822090 | secF preprotein translocase subunit SecF |
|  |  |  | HVO_1976 | 1822087 | 1823661 | secD preprotein translocase subunit SecD |
|  |  |  | HVO_1977 | 1823943 | 1824173 | conserved hypothetical protein |
|  |  |  | HVO_1978 | 1824240 | 1824887 | rnhB ribonuclease HII |
|  |  |  | HVO_1979 | 1824976 | 1826259 | conserved hypothetical protein TIGR01213 |
|  |  |  | HVO_1980 | 1826450 | 1827427 | putative integral membrane protein (TBD) |
|  |  |  | HVO_1981 | 1827568 | 1828161 | hypothetical protein |
|  |  |  | HVO_1982 | 1828262 | 1828555 | expressed protein |
|  |  |  | HVO_1983 | 1829072 | 1830343 | aceB1 malate synthase |
|  |  |  | HVO_1984 | 1830366 | 1831403 | aceA isocitrate lyase |
|  |  |  | HVO_1985 | 1832000 | 1832953 | ABC-type transport system ATP-binding protein |
|  |  |  | HVO_1986 | 1832950 | 1833735 | ABC-type transport system permease protein |
|  |  |  | HVO_1987 | 1833881 | 1834780 | edp proteinase IVlike |
|  |  |  | HVO_1988 | 1834767 | 1835816 | conserved hypothetical protein |
|  |  |  | HVO_1989 | 1836031 | 1836627 | Uncharacterized conserved protein |
|  |  |  | HVO_1990 | 1836712 | 1836784 | tRNAPseudo4 |
|  |  |  | HVO_1991 | 1837331 | 1838560 | ABC-type transport system periplasmic substrate-binding protein (probable substrate iron-III) |
|  |  |  | HVO_1992 | 1838678 | 1838872 | cspD4 cold shock protein |
|  |  |  | HVO_1993 | 1839125 | 1839742 | conserved hypothetical protein |
|  |  |  | HVO_1994 | 1839773 | 1840489 | membrane protein putative |
|  |  |  | HVO_1995 | 1840609 | 1841340 | hypothetical protein |
|  |  |  | HVO_1996 | 1841386 | 1841982 | hydrolase NUDIX family domain protein |
|  |  |  | HVO_1997 | 1842074 | 1842847 | CAAX amino terminal protease family transmembrane |
|  |  |  | HVO_1998 | 1842896 | 1844767 | arsB3 arsenite transport protein |
|  |  |  | HVO_1999 | 1844851 | 1846359 | htr7 transducer protein Htr7 |
|  |  |  | HVO_2000 | 1846356 | 1846637 | conserved hypothetical protein |
|  |  |  | HVO_2001 | 1846826 | 1848376 | tgtA1 queuine/archaeosine tRNA-ribosyltransferase |
|  |  |  | HVO_2002 | 1848449 | 1848724 | putative DNA binding |
|  |  |  | HVO_2003 | 1848816 | 1849109 | conserved hypothetical protein |
|  |  |  | HVO_2004 | 1849160 | 1850023 | serine acetyltransferase |
|  |  |  | HVO_2005 | 1850066 | 1850212 | hypothetical protein |
|  |  |  | HVO_2006 | 1850300 | 1852315 | hypothetical protein |
|  |  |  | HVO_2007 | 1853232 | 1853693 | conserved hypothetical protein |
| **2799** | **2819** | **10D2/470** |  | **1853500** | **1873500** | **Heat shock region** |
|  |  |  | HVO_2008 | 1853807 | 1855564 | tgtA2 queuine/archaeosine tRNA-ribosyltransferase |
|  |  |  | HVO_2009 | 1855802 | 1856071 | hypothetical protein |
|  |  |  | HVO_2010 | 1856174 | 1856482 | conserved hypothetical protein |
|  |  |  | HVO_2011 | 1856667 | 1857056 | conserved hypothetical protein |
|  |  |  | HVO_2012 | 1858184 | 1858579 | putative light and redox sensing histidine kinase |
|  |  |  | HVO_2013 | 1858707 | 1859891 | ftsZ7 cell division protein FtsZ |
|  |  |  | HVO_2014 | 1860015 | 1860512 | conserved hypothetical protein |
|  |  |  | HVO_2015 | 1860594 | 1861631 | conserved hypothetical protein |
|  |  |  | HVO_2016 | 1861770 | 1862669 | putative permease |
|  |  |  | HVO_2017 | 1862777 | 1864156 | conserved hypothetical protein |
|  |  |  | HVO_2018 | 1864256 | 1864615 | hypothetical protein |
|  |  |  | HVO_2019 | 1864886 | 1865158 | PRCbarrel domain putative |
|  |  |  | HVO_2020 | 1865741 | 1866544 | Protein of unknown function (DUF502) family |
|  |  |  | HVO_2021 | 1866807 | 1867007 | hypothetical protein |
|  |  |  | HVO_2022 | 1867278 | 1867883 | hypothetical protein |
|  |  |  | HVO_2023 | 1867948 | 1868175 | hypothetical protein |
|  |  |  | HVO_2024 | 1868835 | 1870061 | hypothetical protein |
|  |  |  | HVO_2025 | 1870301 | 1871404 | HTRlike protein |
|  |  |  | HVO_2026 | 1871507 | 1871686 | hypothetical protein |
|  |  |  | HVO_2027 | 1871717 | 1872109 | terminal quinol oxidase subunit |
|  |  |  | HVO_2028 | 1872299 | 1872502 | lipoprotein putative |
|  |  |  | HVO_2030 | 1872530 | 1874488 | HTRlike protein |
|  |  |  | HVO_2029 | 1874485 | 1874946 | trh5 transcription regulator AsnC family |
|  |  |  | HVO_2031 | 1875211 | 1876422 | tsgA12 ABC-type transport system periplasmic substrate-binding protein (probable substrate sugar) |
|  |  |  | HVO_2032 | 1876480 | 1878048 | tsgD12 ABC-type transport system ATP-binding protein (probable substrate sugar) |
|  |  |  | HVO_2033 | 1878045 | 1879175 | tsgC12 ABC-type transport system permease protein (probable substrate sugar) |
|  |  |  | HVO_2034 | 1879172 | 1880227 | tsgB12 ABC-type transport system permease protein (probable substrate sugar) |
|  |  |  | HVO_2035 | 1880343 | 1881461 | transcription regulator |
|  |  |  | HVO_2036 | 1881529 | 1881720 | conserved hypothetical protein |
|  |  |  | HVO_2037 | 1882056 | 1882334 | hypothetical protein (TBD) |
|  |  |  | HVO_2037_A | 1882817 | 1883035 | conserved hypothetical protein |
|  |  |  | HVO_2038 | 1883147 | 1884325 | ABC-type transport system periplasmic substrate-binding protein (probable substrate iron-III) |
|  |  |  | HVO_2039 | 1884634 | 1885044 | hypothetical protein |
|  |  |  | HVO_2040 | 1885450 | 1886370 | galE4 UDP-glucose 4-epimerase |
|  |  |  | HVO_2041 | 1886684 | 1887007 | hypothetical protein |
|  |  |  | HVO_2042 | 1888354 | 1889583 | orc4 Orc1-type DNA replication protein |
|  |  |  | HVO_2043 | 1890204 | 1890875 | glycosyltransferase family 1 |
|  |  |  | HVO_2044 | 1890935 | 1891897 | I-endH intein-related probable LAGLIDADG endonuclease I-EndH |
|  |  |  | HVO_2045 | 1892176 | 1892412 | hypothetical protein |
|  |  |  | HVO_2046 | 1892437 | 1893654 | Nacetylgalactosamine4sulfatase. putative |
|  |  |  | HVO_2047 | 1893837 | 1894727 | conserved hypothetical protein |
| **50** | **100** | **261 (B40)** |  | **1952250** | **2002250** | **Transcription increased at high salinity, decreased at low salinity** |
|  |  |  | HVO_2088 | 1950195 | 1952432 | xloA xylosidase/arabinosidase |
|  |  |  | HVO_2089 | 1952583 | 1952957 | conserved hypothetical protein |
|  |  |  | HVO_2090 | 1953552 | 1953707 | mandelate racemase/muconate lactonizing enzyme family protein (nonfunctional) |
|  |  |  | HVO_2090_A | 1953705 | 1953923 | IS1341-type transposase (nonfunctional) |
|  |  |  | HVO_2091 | 1954168 | 1955493 | gabT2 4-aminobutyrate aminotransferase |
|  |  |  | HVO_2092 | 1955740 | 1956543 | arcR2 ArcR family transcription regulator |
|  |  |  | HVO_2093 | 1956917 | 1958386 | nhaC4 Na+/H+ antiporter NhaC |
|  |  |  | HVO_2094 | 1958400 | 1959647 | ABC-type transport system periplasmic substrate-binding protein (probable substrate iron-III) |
|  |  |  | HVO_2095 | 1959773 | 1960786 | putative nadpdependent oxidoreductase yncb. |
|  |  |  | HVO_2096 | 1960956 | 1961600 | conserved protein YhfK |
|  |  |  | HVO_2097 | 1961684 | 1962892 | sugar transporter |
|  |  |  | HVO_2098 | 1963002 | 1963151 | hypothetical protein |
|  |  |  | HVO_2099 | 1963459 | 1964253 | putative dioxygenase |
|  |  |  | HVO_2100 | 1964595 | 1964771 | hypothetical protein |
|  |  |  | HVO_2101 | 1964788 | 1965069 | ptsH3 phosphocarrier protein HPr |
|  |  |  | HVO_2102 | 1965302 | 1965763 | PTS system galactitolspecific enzyme II A component putative |
|  |  |  | HVO_2103 | 1965879 | 1967369 | PTS system galactitolspecific enzyme II C component |
|  |  |  | HVO_2104 | 1967493 | 1967789 | presumptive arabitol PTS enzyme IIB |
|  |  |  | HVO_2105 | 1967896 | 1968573 | tpiA2 triosephosphate isomerase |
|  |  |  | HVO_2106 | 1968602 | 1969378 | fbaB Fructose1 6bisphosphate aldolase of the DhnA family |
|  |  |  | HVO_2107 | 1969381 | 1969806 | conserved hypothetical protein TIGR00149 |
|  |  |  | HVO_2108 | 1970018 | 1970779 | arcR3 ArcR family transcription regulator |
|  |  |  | HVO_2109 | 1971127 | 1972587 | Xylulose kinase putative |
|  |  |  | HVO_2110 | 1973308 | 1974072 | arcR4 ArcR family transcription regulator |
|  |  |  | HVO_2111 | 1974142 | 1975152 | pdxA 4hydroxythreonine4phosphate dehydrogenase |
|  |  |  | HVO_2112 | 1975149 | 1976387 | ygbK domain protein |
|  |  |  | HVO_2113 | 1976493 | 1977920 | tsgA2 ABC-type transport system periplasmic substrate-binding protein (probable substrate sugar) |
|  |  |  | HVO_2114 | 1978054 | 1978989 | Dihydrodipicolinate synthase putative |
|  |  |  | HVO_2115 | 1979250 | 1980047 | oxidoreductase aldo/keto reductase family |
|  |  |  | HVO_2116 | 1980207 | 1981151 | tsgB2 ABC-type transport system permease protein (probable substrate sugar) |
|  |  |  | HVO_2117 | 1981148 | 1982068 | tsgC2 ABC-type transport system permease protein (probable substrate sugar) |
|  |  |  | HVO_2118 | 1982189 | 1983292 | tsgD2 ABC-type transport system ATP-binding protein (probable substrate sugar) |
|  |  |  | HVO_2119 | 1983304 | 1984137 | conserved hypothetical protein |
|  |  |  | HVO_2120 | 1984201 | 1985358 | BNR/Aspbox repeat domain protein |
|  |  |  | HVO_2121 | 1985891 | 1986100 | hypothetical protein |
|  |  |  | HVO_2122 | 1986097 | 1987203 | dppF4 ABC-type transport system ATP-binding protein (probable substrate dipeptides/oligopeptides) |
|  |  |  | HVO_2123 | 1987200 | 1988276 | dppD4 ABC-type transport system ATP-binding protein (probable substrate dipeptides/oligopeptides) |
|  |  |  | HVO_2124 | 1988273 | 1989277 | dppC4 ABC-type transport system permease protein (probable substrate dipeptides/oligopeptides) |
|  |  |  | HVO_2125 | 1989279 | 1990238 | dppB4 ABC-type transport system permease protein (probable substrate dipeptides/oligopeptides) |
|  |  |  | HVO_2126 | 1990314 | 1992035 | dppA4 ABC-type transport system periplasmic substrate-binding protein (probable substrate dipeptides/oligopeptides) |
|  |  |  | HVO_2127 | 1992248 | 1993510 | indole3acetylL aspartic acid hydrolase |
|  |  |  | HVO_2128 | 1993640 | 1994896 | amaB1 N-carbamyl-L-cysteine amidohydrolase |
|  |  |  | HVO_2129 | 1994893 | 1995492 | hypothetical protein |
|  |  |  | HVO_2130 | 1995516 | 1996334 | arcR5 ArcR family transcription regulator |
|  |  |  | HVO_2131 | 1996962 | 1997831 | fbp2 fructose-1,6-bisphosphatase |
|  |  |  | HVO_2132 | 1998144 | 1998317 | hypothetical protein |
|  |  |  | HVO_2133 | 1998477 | 1999643 | orc16 Orc1-type DNA replication protein |
|  |  |  | HVO_2134 | 1999777 | 2000010 | Protein |
|  |  |  | HVO_2135 | 2000143 | 2001483 | conserved hypothetical protein |
|  |  |  | HVO_2136 | 2001515 | 2001814 | conserved hypothetical protein |
|  |  |  | HVO_2137 | 2001973 | 2002281 | Cupin domain protein |
| **348** | **365** | **RE-416** |  | **2250257** | **2267257** | **Expressed strongly in rich medium conditions** |
|  |  |  | HVO_2383 | 2249984 | 2250673 | radB DNA repair and recombination protein RadB |
|  |  |  | HVO_2384 | 2250787 | 2251929 | CBS domain pair putative |
|  |  |  | HVO_2385 | 2252319 | 2254595 | pilB5 type IV pilus biogenesis complex ATPase subunit |
|  |  |  | HVO_2386 | 2254585 | 2256711 | pilC5 type IV pilus biogenesis complex membrane subunit |
|  |  |  | HVO_2387 | 2256716 | 2258887 | conserved hypothetical protein |
|  |  |  | HVO_2388 | 2258891 | 2260621 | predicted protein |
|  |  |  | HVO_2389 | 2260741 | 2261445 | dehII haloacid dehalogenase type II |
|  |  |  | HVO_2390 | 2261527 | 2261898 | hypothetical protein |
|  |  |  | HVO_2391 | 2261991 | 2262941 | conserved hypothetical protein |
|  |  |  | HVO_2392 | 2263590 | 2263778 | conserved hypothetical protein |
|  |  |  | HVO_2393 | 2264001 | 2264075 | tRNAVal2 |
|  |  |  | HVO_2394 | 2264147 | 2264398 | conserved hypothetical protein |
|  |  |  | HVO_2395 | 2264658 | 2265191 | ATP cob(I)alamin adenosyltransferase putative |
|  |  |  | HVO_2396 | 2265188 | 2265427 | glutaredoxinlike |
|  |  |  | HVO_2397 | 2265616 | 2266722 | znuA1 ABC-type transport system periplasmic substrate-binding protein (probable substrate zinc) |
|  |  |  | HVO_2398 | 2266719 | 2267588 | znuC1 ABC-type transport system ATP-binding protein (probable substrate zinc) |
| **450** | **460** | **531 (D57)** |  | **2352250** | **2362250** | **Transcribed slightly at high salinity** |
|  |  |  | HVO_2484 | 2352390 | 2352956 | uspA22 uspA domain protein |
|  |  |  | HVO_2485 | 2352996 | 2354000 | biotinacetylCoAcarboxylase ligase |
|  |  |  | HVO_2486 | 2354069 | 2355868 | bccA biotin carboxylase |
|  |  |  | HVO_2487 | 2356066 | 2357100 | asd aspartatesemialdehyde dehydrogenase |
|  |  |  | HVO_2488 | 2357537 | 2357734 | conserved hypothetical protein |
|  |  |  | HVO_2489 | 2358034 | 2358303 | conserved hypothetical protein |
|  |  |  | HVO_2490 | 2358413 | 2358739 | hypothetical protein |
|  |  |  | HVO_2491 | 2358758 | 2358828 | tRNAPseudo5 |
|  |  |  | HVO_2492 | 2359002 | 2359286 | hypothetical protein |
|  |  |  | HVO_2493 | 2359296 | 2360165 | tRNA pseudouridine synthase B |
|  |  |  | HVO_2494 | 2360168 | 2360746 | cytidylate kinase putative |
|  |  |  | HVO_2495 | 2360965 | 2361882 | htlB HTRlike protein |
| **459** | **464** | **531/D57** |  | **2361257** | **2366257** | **Heat shock region and expressed more strongly in Chemically Defined Medium** |
| **460** | **480** | **266/D57** |  | **2362250** | **2382250** | **Transcribed at high salinity** |
|  |  |  | HVO_2496 | 2362033 | 2362659 | adk Adenylate kinase |
|  |  |  | HVO_2497 | 2363011 | 2364030 | membrane protein putative |
|  |  |  | HVO_2498 | 2364122 | 2364517 | conserved hypothetical protein |
|  |  |  | HVO_2499 | 2364709 | 2365179 | uspA23 uspA domain protein |
|  |  |  | HVO_2500 | 2365176 | 2367422 | cat2 cationic amino acid transporter |
|  |  |  | HVO_2501 | 2367604 | 2368554 | epoxide hydrolase homolog yfhM |
|  |  |  | HVO_2502 | 2368893 | 2369933 | hmf hydrogenase expression/formation protein |
|  |  |  | HVO_2503 | 2369988 | 2371265 | kynU kynureninase |
|  |  |  | HVO_2504 | 2371290 | 2371988 | atsC 3oxoacyl acylcarrier protein reductase |
|  |  |  | HVO_2505 | 2372097 | 2372516 | Mov34MPNPAD1 superfamily |
|  |  |  | HVO_2506 | 2372503 | 2373072 | Isopentenyldiphosphate deltaisomerase |
|  |  |  | HVO_2507 | 2373145 | 2373558 | trh transcription regulator |
|  |  |  | HVO_2508 | 2373656 | 2374738 | carA carbamoylphosphate synthase small subunit |
|  |  |  | HVO_2509 | 2374932 | 2375969 | putative light and oxygensensing transcription regulator |
|  |  |  | HVO_2510 | 2376033 | 2376947 | gnat family acetyltransferase |
|  |  |  | HVO_2511 | 2376952 | 2378199 | gatD glutamyl-tRNA(Gln) amidotransferase subunit D |
|  |  |  | HVO_2512 | 2378431 | 2378640 | membrane protein |
|  |  |  | HVO_2513 | 2378737 | 2379234 | conserved hypothetical protein |
|  |  |  | HVO_2514 | 2379238 | 2379891 | conditioned mediuminduced protein 2 |
|  |  |  | HVO_2515 | 2379957 | 2380658 | membrane protein putative |
|  |  |  | HVO_2516 | 2380794 | 2382374 | gpmI 2 3bisphosphoglycerateindependent phosphoglycerate mutase |
| **624** | **644** | **H11** |  | **2526257** | **2546257** | **Heat shock region** |
|  |  |  | HVO_2675 | 2525128 | 2526414 | hisD histidinol dehydrogenase |
|  |  |  | HVO_2676 | 2526511 | 2526876 | conserved hypothetical protein putative |
|  |  |  | HVO_2677 | 2526916 | 2527164 | acetyltransferase (gnat) family |
|  |  |  | HVO_2678 | 2527253 | 2527597 | conserved hypothetical protein |
|  |  |  | HVO_2679 | 2527654 | 2528397 | membrane protein putative |
|  |  |  | HVO_2680 | 2528394 | 2529053 | conserved hypothetical protein |
|  |  |  | HVO_2681 | 2529154 | 2529951 | conserved hypothetical protein |
|  |  |  | HVO_2682 | 2530045 | 2530257 | dodecinrelated protein |
|  |  |  | HVO_2683 | 2530375 | 2531754 | 2'3'cyclicnucleotide 2'phosphodiesterase |
|  |  |  | HVO_2684 | 2531839 | 2532246 | conserved hypothetical protein |
|  |  |  | HVO_2685 | 2532346 | 2533494 | transporter small conductance mechanosensitive ion channel (MscS) family family |
|  |  |  | HVO_2686 | 2533589 | 2533798 | conserved hypothetical protein |
|  |  |  | HVO_2687 | 2533835 | 2534329 | conserved hypothetical protein |
|  |  |  | HVO_2688 | 2534459 | 2535520 | sugarspecific transcriptional regulator TrmB |
|  |  |  | HVO_2689 | 2535581 | 2536375 | conserved hypothetical protein |
|  |  |  | HVO_2690 | 2536418 | 2537518 | oxidoreductase |
|  |  |  | HVO_2691 | 2537673 | 2537876 | conserved hypothetical protein |
|  |  |  | HVO_2692 | 2537992 | 2539179 | tsgD3 ABC-type transport system ATP-binding protein (probable substrate sugar) |
|  |  |  | HVO_2693 | 2539182 | 2540141 | tsgC3 ABC-type transport system permease protein (probable substrate sugar) |
|  |  |  | HVO_2694 | 2540143 | 2541168 | tsgB3 ABC-type transport system permease protein (probable substrate sugar) |
|  |  |  | HVO_2695 | 2541219 | 2542757 | tsgA3 ABC-type transport system periplasmic substrate-binding protein (probable substrate sugar) |
|  |  |  | HVO_2696 | 2542912 | 2543889 | oxidoreductase aldo/keto reductase family |
|  |  |  | HVO_2697 | 2544015 | 2545172 | priS DNA primase eukaryotictype small subunit putative |
|  |  |  | HVO_2698 | 2545169 | 2546164 | ginS DNA replication factor GINS |
|  |  |  | HVO_2699 | 2546201 | 2546659 | bcp1 peroxiredoxin |
